# Using optical coherence tomography and optical coherence tomography angiography to delineate neurovascular homeostasis in migraine: a review

**DOI:** 10.3389/fnins.2024.1376282

**Published:** 2024-04-15

**Authors:** Devahuti R. Chaliha, Mauro Vaccarezza, Jason Charng, Fred K. Chen, Amy Lim, Peter Drummond, Ryusuke Takechi, Virginie Lam, Satvinder S. Dhaliwal, John C. L. Mamo

**Affiliations:** ^1^Faculty of Health Sciences, Curtin Health Innovation Research Institute, Curtin University, Perth, WA, Australia; ^2^Faculty of Health Sciences, School of Population Health, Curtin University, Perth, WA, Australia; ^3^Faculty of Health Sciences, Curtin Medical School, Curtin University, Perth, WA, Australia; ^4^Centre for Ophthalmology and Visual Sciences (Lions Eye Institute), The University of Western Australia, Perth, WA, Australia; ^5^Department of Optometry, School of Allied Health, The University of Western Australia, Perth, WA, Australia; ^6^Ophthalmology, Department of Surgery, University of Melbourne, Melbourne, VIC, Australia; ^7^Centre for Healthy Ageing, Health Futures Institute, Murdoch University, Perth, WA, Australia; ^8^Perron Institute Neurological and Translational Sciences, Perth, WA, Australia; ^9^Duke-NUS Medical School, National University of Singapore, Singapore, Singapore; ^10^Institute for Research in Molecular Medicine (INFORMM), Universiti Sains Malaysia, Gelugor, Pulau Pinang, Malaysia; ^11^Singapore University of Social Sciences, Singapore, Singapore

**Keywords:** optical coherence tomography, optical coherence tomography angiography (OCTA), migraine, retina, choroid, vasculature, vasodilation, vasoconstriction

## Abstract

Migraine is one of the world’s most debilitating disorders, and it has recently been shown that changes in the retina can be a potential biomarker for the disease. These changes can be detected by optical coherence tomography (OCT), which measures retinal thickness, and optical coherence tomography angiography (OCTA), which measures vessel density. We searched the databases Google Scholar, ProQuest, Scopus, and Web of Science for studies in English using OCT and OCTA in migraineurs, using the search terms “optical coherence tomography,” “OCT,” “optical coherence tomography angiography,” “OCTA” and “migraine.” We found 73 primary studies, 11 reviews, and 8 meta-analyses pertaining to OCT and OCTA findings in migraineurs. They showed that migraineurs had reduced retinal thickness (via OCT), retinal vessel density, and greater foveal avascular zone area (via OCTA) than controls. OCTA changes reflect a perfusion compromise occurring in migraineurs as opposed to in healthy controls. OCT and OCTA deficits were worse in migraine-with-aura and chronic migraine than in migraine-without-aura and episodic migraine. Certain areas of the eye, such as the fovea, may be more vulnerable to these perfusion changes than other parts. Direct comparison between study findings is difficult because of the heterogeneity between the studies in terms of both methodology and analysis. Moreover, as almost all case–control studies were cross-sectional, more longitudinal cohort studies are needed to determine cause and effect between migraine pathophysiology and OCT/OCTA findings. Current evidence suggests both OCT and OCTA may serve as retinal markers for migraineurs, and further research in this field will hopefully enable us to better understand the vascular changes associated with migraine, perhaps also providing a new diagnostic and therapeutic biomarker.

## Introduction

OCT measures retinal thickness, employing infra-red wavelength for image acquisition, and has an 8–10 μm axial resolution, which may be improved to 3 μm in some devices (Wojtkowski et al., 2004; Ascaso et al., 2017). Spectral-domain OCT (Ewering et al., 2015; Karalezli et al., 2015; Yu et al., 2016; Ulusoy et al., 2019) provides higher resolution, faster acquisition speed, and fewer artifacts (Forte et al., 2009). However, limitations include a small field of view, lack of vessel leakage detection, dependency of resolution on coherence length of light source, artifacts from minute patient movements, and inability to detect slower blood flow (Huang et al., 1991; De Carlo et al., 2015). Retinal thickness data in OCT images can be partitioned into four (superior, inferior, nasal, and temporal) or six (nasal, superior nasal, inferior nasal, temporal, superior temporal, and inferior temporal) regions, and one meta-analysis showed that the four-region partition method detected superior and inferior quadrant retinal nerve fiber layer (RNFL) thickness differences better than the six-region method for delineating migraine findings (Lin et al., 2021). The RNFL is the layer formed by the retinal ganglion cell axons, which collect visual impulses from the rods and cones of the retina.

Optical coherence tomography angiography (OCTA) is a non-invasive retinal vascular imaging technique generating a 3D image of the layers of retinal vasculature via motion contrast of blood flow (An and Wang, 2008; Mariampillai et al., 2008; De Carlo et al., 2015; Spaide et al., 2015; Campbell et al., 2017; Fujimoto et al., 2023; Toth, 2023). This fast, continuous, repeated longitudinal scanning method allows blood cells inside the vessel lumen to be discriminated from surrounding tissue, and hence blood flow to be tracked scan by scan (De Carlo et al., 2015; Toth, 2023). This technique has several advantages over invasive retinal imaging methods such as fluorescence angiography and indocyanine green angiography, which have adverse effects and contraindications associated with the dye and its injection, as well as superimposed imaging of all layers in the retina owing to a lack of depth resolution and being time-consuming and expensive (De Carlo et al., 2015; Spaide et al., 2015). The compromise between signal bandwidth and detection sensitivity affects the maximum acquisition rate in OCT (Huang et al., 1991), but OCTA may overcome this with greater imaging speeds (De Carlo et al., 2015). It also outperforms fundus photography, which only provides a 2D visualization of blood vessels with low resolution (Chang et al., 2017).

Tomography involves taking cross-sectional images of 3D objects, and in 1991, Huang, Swanson, and Fujimoto used the coherence properties of light waves to apply the technique to a human eye *in vitro* (Huang et al., 1991). This can now be used via ophthalmoscope and camera *in vivo*, whereas previous eye-imaging methods could only take place on fixed tissue (Toth, 2023). Since the first publication of this innovative technique in 1991 (Huang et al., 1991), optical coherence tomography (OCT) has been used in several medical fields with an increasing recognition that stems from its properties and potential clinical applications (Fujimoto et al., 2023; Toth, 2023; Tzaridis and Friedlander, 2023).

OCTA, on the other hand, provides a robust assessment of the retinal vasculature, and it can also discriminate between superficial and deep capillary plexus networks (Magrath et al., 2017). OCTA can precisely show capillaries undergoing ischemia (Pang et al., 2023). OCTA is superior to traditional methods for imaging radial pericapillary and deep capillary networks based on flow characteristics (De Carlo et al., 2015; Spaide et al., 2015), and non-perfusion can therefore be quantified accurately (Campbell et al., 2017) with a high data acquisition rate (Huang et al., 1991). In fact, projection-resolved (PR) OCTA improves on conventional OCT by addressing the problem of the superficial vessels projecting flow artifacts detected from the deeper layers, therefore enhancing depth resolution (Campbell et al., 2017).

Nowadays, OCTA is widely used in ophthalmology and cardiovascular disease as a powerful tool called an “optical biopsy” (Brezinski et al., 1996; Tearney et al., 1997; Fujimoto et al., 2023). The retina is the only vasculature that can be visualized non-invasively *in vivo* (Pang et al., 2023). We consider the eye as an extension of the brain because the optic nerve, retina, and brain derive from the anterior neural tube during embryonic development (London et al., 2013). The retina and cortex have similar angiogenesis patterns in development (Chan-Ling et al., 2004), so the brain and retina have close and similar blood regulations (Reiner et al., 2018).

One of the vascular-derived disorders that can be assessed via OCT/OCTA is migraine. Migraine incidence worldwide was 87.6 million in 2019 (Fan et al., 2023). Episodic migraine (<15 migraine days per month) is believed to originate from neuronal hyperexcitability in the trigeminal vascular system (Welch et al., 2003) and can transform to chronic (15 or more days/month) through increased attack frequencies, perhaps due to functional and structural brain changes, central sensitization and neuroinflammation (Mungoven et al., 2021). Migraine can occur with or without aura, where aura occurs in 15% of migraineurs from the cortical spreading depression preceding a migraine attack. Aura symptoms are sensory disturbances, with 90% being visual, associated with more severe ischemic risk (Lucas, 2021).

**Figure 1:**
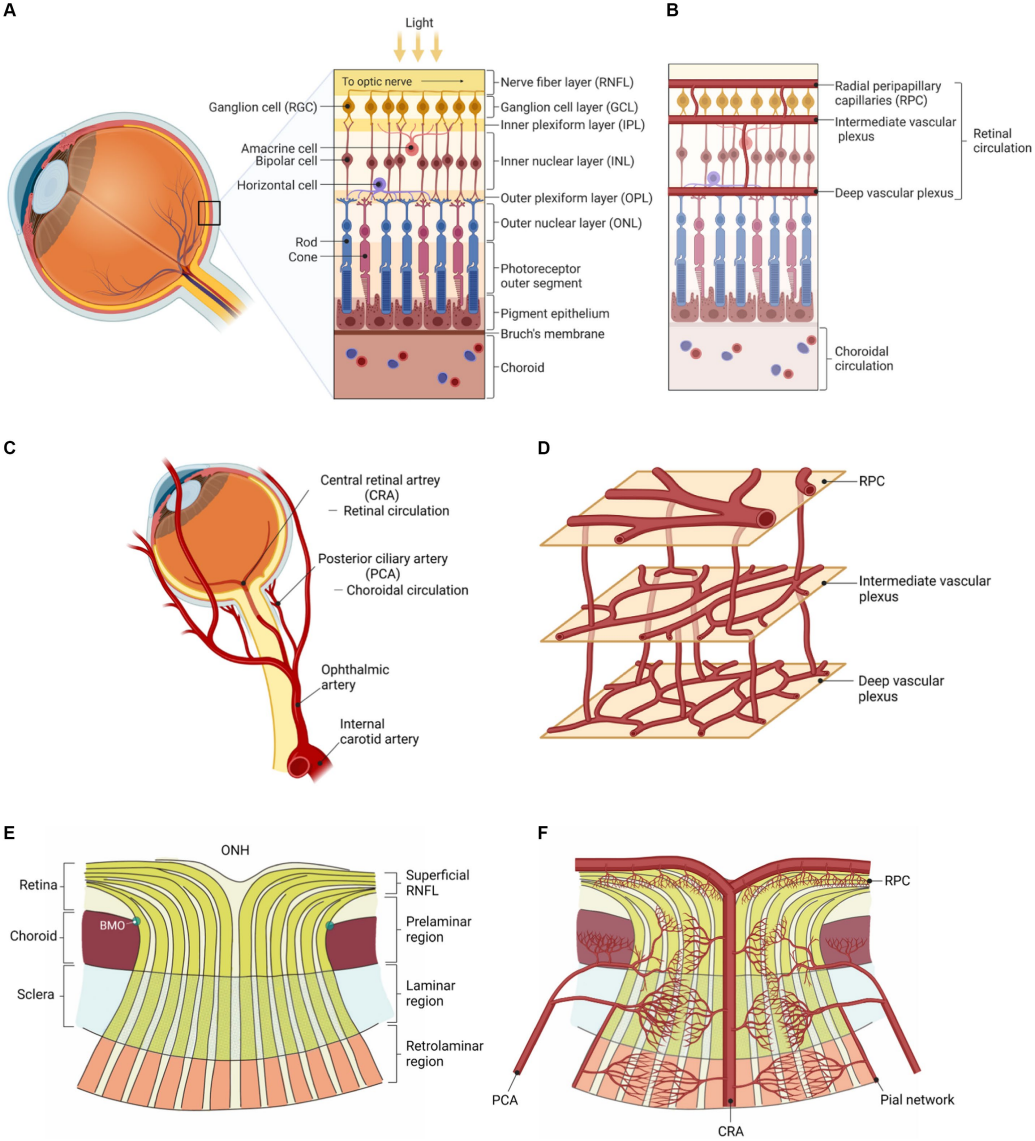
Vascular and neural anatomy of the eye (Shiga et al., 2023). **(A)** Neuronal layers of the retina. **(B)** Vascular layers of the retina. **(C)** Retinal (supplying the inner retina) vs. choroidal (supplying the outer retina) circulations. **(D)** The three interconnected and anastomosing vascular plexuses of the retina. **(E)** Neuronal layers of the eye converging into the optic nerve (ONH: optic nerve head). **(F)** Vascular layers of the eye branching out from the central retinal artery (at the ONH). Adapted from Shiga et al. (2023) reproduced under the terms of CC BY 4.0.

It is believed that migraine involves chronic systemic vasoconstriction, with (Chang et al., 2017) and without aura (Komatsu et al., 2003). We previously hypothesized that heightened sympathetic tone results in progressive central microvascular constriction. Suboptimal parenchymal blood flow may activate nociceptors and trigger the migraine (Chaliha et al., 2020). This may be seen in the fundus as reduced vessel density. Vessel density can be measured as blood vessel length divided by scan area (Chang et al., 2017) or the percentage of vascularized tissue within the area (Taşlı and Ersoy, 2020). From this, several areas of the posterior eye can be analyzed using OCTA. A schematic of the eye, showing vascular and neural anatomy, is shown in Figure 1 (Shiga et al., 2023).

### Aim/background

The current literature regarding cerebral and retinal vascular perfusion suggests that retinal vascular changes can indicate cerebral vascular disease, even proportionally, due to the common embryological origin and resultant homology between the retinal and cerebral microvasculatures (Patton et al., 2005; Moss, 2015). For example, retinopathy signs such as retinal artery occlusion or greater retinal vein caliber may indicate cerebrovascular compromise (Moss, 2015). In recent studies, lower retinal perfusion has been associated with MRI biomarkers of cerebral small-vessel disease (Wang et al., 2021; Abdolahi et al., 2023). Previous studies examining migraine patients using OCT and OCTA show several differences between migraineurs (also with vs. without aura) and healthy controls in vascular tone in the retinal macular and retinal optic nerve areas, as measured via vessel density as well as choroidal and retinal thicknesses. This particular review highlights the relevance such OCT/OCTA signs have for the severity, classification, and prediction of migraine episodes. The purpose of this study was to collate the information from various OCTA studies pertaining to migraine and sort them into ocular structural categories, as has not been done before to our knowledge. In the anterior eye, there seemed to be no differences in axial length, corneal curvature radius, anterior chamber depth, central corneal thickness, or pupil size between migraineurs and controls, ictally or interictally (Koban et al., 2016). Hence, we will focus only on the retinal and choroidal layers of the eye. For this review, we found different studies using OCT and OCTA to investigate migraineurs, using the search terms “optical coherence tomography,” “OCT,” “optical coherence tomography angiography,” “OCTA,” and “migraine” present in the abstracts and sorted by relevance on the databases Google Scholar, ProQuest, Scopus, and Web of Science via institutional access, as well as the reference lists of all the OCT/OCTA-migraine systematic reviews and meta-analyses we found. Only articles in English or with available English translations have been included. Our results are presented in Supplementary Table S1.

## Retina

Vessel density (VD) is taken as the length/area of flowing vessels as a percentage of the total area scanned (Pang et al., 2023) or the percentage area of vessels with active (OCTA-detectable) blood flow (Ke et al., 2022). The deep capillary network comprises multi-capillary units converging toward a central vortex of capillaries draining to superficial venules, which OCTA is able to detect (Bonnin et al., 2015). Both our search and Pang’s nine-study meta-analysis show that migraineurs had lower superficial plexus, deep plexus, macular, peripapillary (area around the retinal papillary region), and foveal VD than controls (Güler et al., 2020; Pang et al., 2023). Often, these reductions were present in those with aura only, although there was a similar tendency in non-aura migraineurs.

Aura and chronic migraineurs seem to have greater reductions than non-aura and episodic migraineurs. Aura migraineurs especially tended to have decreased superficial, deep, and parafoveal deep capillary plexus VD than did controls (Ke et al., 2022). Participants with a history of migraines with aura show lower retinal arteriole caliber compared to controls with no migraine history (Liew et al., 2006). In chronic but not episodic migraineurs, retinal arteries were bulkier ipsilaterally to the headache compared to controls (Unlu et al., 2017), but this did not correspond with retinal vein diameters, which were similar (Unlu et al., 2017). This suggests greater increased energy demand at the retina in chronic migraineurs. Unsurprisingly, migraineurs, especially those with aura, seem to have retinal capillary damage (Liu et al., 2023).

During migraine attacks and/or auras, transient vasospasm can compromise perfusion in both the eye and head (Liu et al., 2023). This can change retinal/neuronal perfusion, in turn causing hypoxic/ischemic injury, ultimately damaging the retinal nerve (Liu et al., 2023) and structures. Lower deep retinal VD is correlated with lower signal strength index, longer axial length, and higher creatinine, where creatinine acts as an energy buffer for retinal cells (Tachikawa et al., 2007; You et al., 2019). OCTA seems to be able to detect these changes, but there may be confounders. Lower superficial retinal VD is correlated with lower signal strength index and participant sex being male (You et al., 2019). This may underscore the importance of keeping male/female proportions similar between groups.

On OCT, retinal thickening may indicate oedema, whereas retinal thinning may indicate atrophy (Toth, 2023). It can be split into looking at the retinal nerve fiber layer (RNFL) and the ganglion cell layer (GCL). The OCT signal originates from two plexuses in the inner retina (SCP and DCP), both of which seem to be affected by migraine pathophysiology.

### Retinal nerve fiber layer (RNFL)

**Figure 2:**
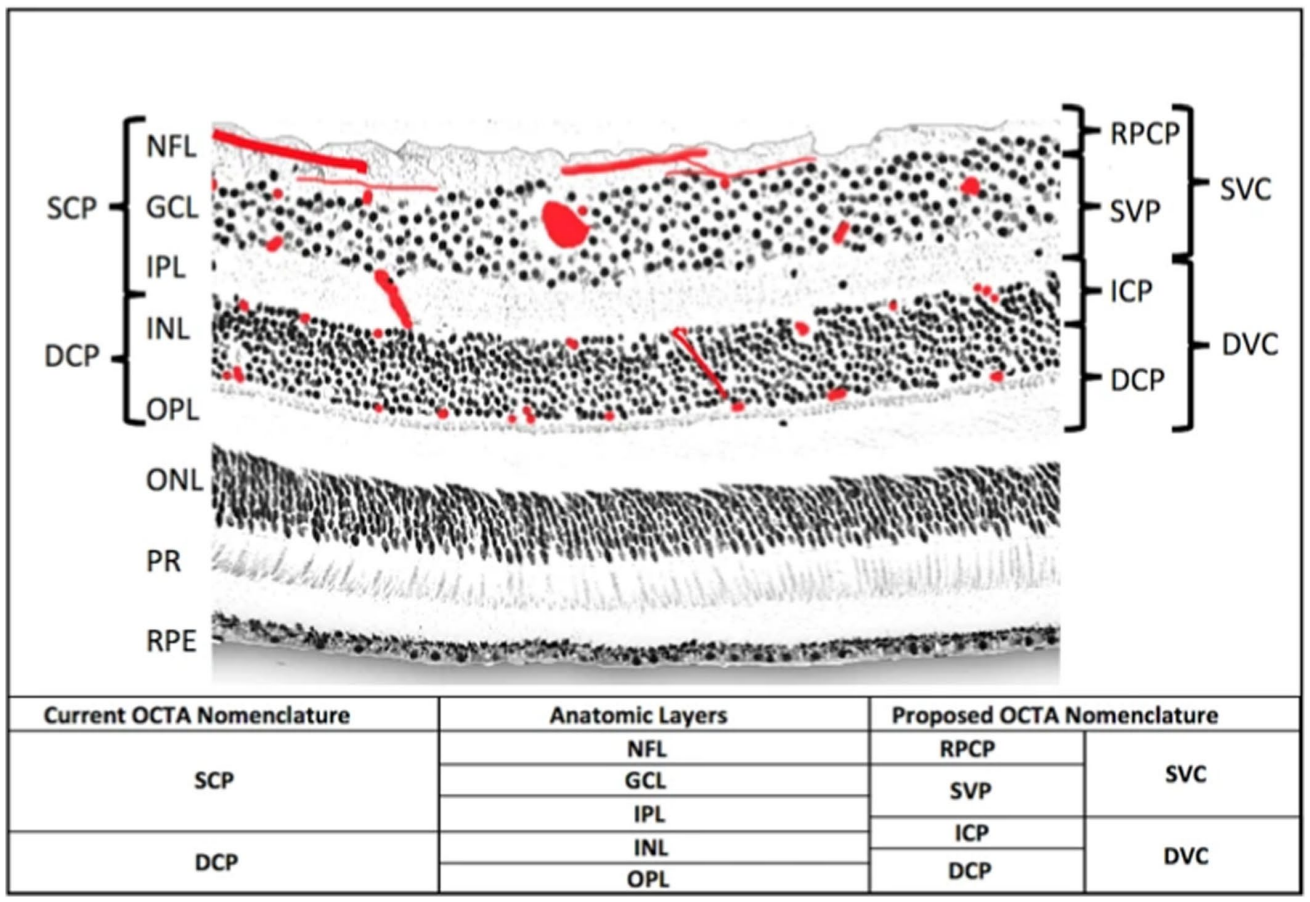
An histological cross-section of the retina (Campbell et al., 2017). The red blots represent vascular plexuses. DCP: deep capillary plexus; DVC: deep vascular complex; GCL: ganglion cell layer; ICP: intermediate capillary plexus; INL: inner nuclear layer; IPL: inner plexiform layer; NFL: nerve fibre layer; ONL: outer nuclear layer; OPL: outer plexiform layer plus Henle’s fibre layer; PR: photoreceptor layers; RPCP: radial peripapillary capillary plexus; RPE: retinal pigment epithelium; SCP: superficial capillary plexus; SVC: superficial vascular complex; SVP: superficial vascular plexus. Adapted from Campbell et al. (2017) under the terms of CC BY 4.0.

Retinal thickness can also perhaps be examined to indicate perfusion related to migraines, and OCT allows high-resolution RNFL, GCL, and choroid thickness determination *in vivo* (Ascaso et al., 2017). RNFL thinning is actually associated with brain atrophy in general, with direct correlations with the central cingulate and pericalcarine cortices, especially in certain neurodegenerative diseases (Von Glehn et al., 2014; Shi et al., 2019). OCT can be used to measure the thickness of the RNFL, usually in the peripapillary (optic nerve head) region and macula (Ascaso et al., 2017). In controls, the inner retinal layers’ thickness correlates with retinal microvascular perfusion when using OCTA to visualize macular and peripapillary areas (Yu et al., 2016). On OCTA, vessel density in the RNFL is another measure of perfusion, and predictably, in some studies, investigators found that aura migraineurs had lower VD there than controls and that the more the migraine frequency/disability/history, the lower the VD (Hamamci et al., 2021; Hamurcu et al., 2021). Figure 2 shows a histology section showing the two capillary plexuses from which OCTA signals are derived (Campbell et al., 2017). Out of the superficial vascular complex (SVC), it should be noted that the radial peripapillary capillary plexus (RPCP) is found predominantly adjacent to the optic nerve, and the superficial vascular plexus (SVP) is found predominantly in the macula. The SVP density and GCL thickness decrease away from the optic nerve (Campbell et al., 2017).

We found 35 studies examining specifically the RNFL in migraineurs and found that studies have inconsistently shown that migraineurs have thinner RNFL than controls (Cunha et al., 2008; Costello, 2009; Gipponi et al., 2013; Sorkhabi et al., 2013; Ekinci et al., 2014; Demircan et al., 2015; Yülek et al., 2015; Acer et al., 2016; Demirci et al., 2016; Feng et al., 2016; Verroiopoulos et al., 2016; Ergiyit, 2017; Reggio et al., 2017; Tunç et al., 2017; Abdellatif and Fouad, 2018; Tak et al., 2018; Bing et al., 2019; Ulusoy et al., 2019; You et al., 2019; Sirakaya et al., 2020; Yener and Yılmaz, 2020; Altunisik and Oren, 2021; Kanar et al., 2021; Panicker et al., 2021; Temel et al., 2021; Yurtoğulları et al., 2021), generally ipsilateral to the usual side of headache (Martinez et al., 2008; Gunes et al., 2016; Khosravi et al., 2018). Lin et al. examined 26 studies and also found thinner mean RNFL in migraineurs than in controls, especially in the superior and inferior eye quadrants (Lin et al., 2021). Feng et al. meta-analyzed six studies whose investigators found migraineurs to have a much thinner average RNFL than controls, significant in all four retinal regions (superior, inferior, nasal, and temporal) (Feng et al., 2016). Notably, they did not detect publication bias (Feng et al., 2016), although this may have been difficult to identify as they tested fewer than 10 studies.

RNFL thinning indicates axon loss (Martinez et al., 2008). It could serve as a marker for hypoxic damage to ganglion cells and retinal nerve fibers/axons (Gunes et al., 2018; Raga-Martínez et al., 2022), especially given an association between macular RNFL thinning and the anti-oxidant molecular marker catalase (Bulboacă et al., 2020). The longer the migraine history/attack/aura, or the higher the migraine frequency/disability, the thinner the RNFL (Martinez et al., 2008; Costello, 2009; Gipponi et al., 2013; Yülek et al., 2015; Reggio et al., 2017; Ao et al., 2019; Ulusoy et al., 2019; Sirakaya et al., 2020; Dereli Can et al., 2021; Burgos-Blasco et al., 2023). In children, there may not have been time for this effect to develop (Dereli Can et al., 2021). Migraineurs have transient retinal vasospasm ictally, leading to visual aura postdromally (McKendrick and Nguyen, 2022). These recurrent retinal vasospasms may chronically lead to permanent vascular changes in the eye, detectable via OCT (McKendrick and Nguyen, 2022). The recurring transient retinal and ciliary artery constrictions may cause hypoxic damage to the optic nerve, retina, and choroid; thereby, the RNFL and GCL may undergo thinning with general retinal capillary decrement (Xue, 2022).

Migraine-related disability and average RNFL thinning may be strongly correlated through the Migraine Disability Assessment (MIDAS) (Martinez et al., 2008; Sorkhabi et al., 2013), but not through the Visual Analog Scale (VAS) (Yülek et al., 2015), scoring system. Sample heterogeneities may have also played a role in this discrepancy. For example, both eyes, a randomized eye, or only the right or left eye may be selected for the study. A systematic review showed that those choosing only the left eye found no such RNFL thinning, whereas the other studies’ investigators did find RNFL thinning in migraineurs compared to controls (Lin et al., 2021). The random and right-eye selection methods yielded medium effects, whereas both-eye selection yielded small effects (Lin et al., 2021). We wonder if most of the migraineurs had right-sided migraines most of the time. In addition, physiological confounders such as visual aura could have exacerbated RNFL thinning in some studies (Lin et al., 2021).

Aura migraineurs show more severe changes than do non-aura migraineurs. The effect size and amount of thinning were greater in migraineurs with than without aura (Lin et al., 2021). Aura migraineurs had thinner RNFL in many different ocular areas compared to controls and often non-aura migraineurs (Ekinci et al., 2014; Tunç et al., 2017; Labib et al., 2020; Kanar et al., 2021; Lin et al., 2021; Burgos-Blasco et al., 2023; Sim et al., 2023), whereas non-aura migraineurs sometimes did (Tunç et al., 2017) and sometimes did not (Ao et al., 2019; Burgos-Blasco et al., 2023) have significant changes relative to controls. Still, others did not find such differences between aura and non-aura migraineurs (Sorkhabi et al., 2013; Demirci et al., 2016; Nalcacioglu et al., 2017; Yu et al., 2022), especially at the macula (Kirbas et al., 2013; Salman et al., 2015; Simsek et al., 2015; Hamurcu et al., 2021). Aura migraineurs were likelier to have RNFL thinning than non-aura migraineurs, while most auras were visual (Rasmussen and Olesen, 1992; Lin et al., 2021). Indeed, visual aura migraineurs had thinner RNFL than non-visual aura migraineurs in another study (Torun et al., 2023). Aura migraineurs had RNFL thickening interictally but remaining thinner than controls (El-Shazly et al., 2017). Ictally, the posterior hemisphere shows hypoperfusion with ipsilateral aura (Killer et al., 2003), and migraineurs with aura may show greater RNFL changes than those without (Ascaso et al., 2017).

Chronic migraineurs had thinner RNFL than episodic migraineurs (Reggio et al., 2017) and controls (Kirbas et al., 2013; Labib et al., 2020; Raga-Martínez et al., 2022; Oba et al., 2023). Studies show that mainly chronic migraineurs (as opposed to episodic) have thinner retinae and choroids compared to controls, with the RNFL and fovea more affected in those with aura than those without (Ascaso et al., 2017). An acute attack may not affect macular or peripapillary perfusion (Güler et al., 2020). However, RNFL thinning does not seem to be related to migraine duration or history, so perhaps it is not cumulative, but rather, its likelihood of acute occurrence may increase with each acute attack (McKendrick and Nguyen, 2022). Participants with a migraine history less than 15 years had thinner temporal RNFL than controls, but those with more than a 15-year migraine history had thinner average, superior, inferior, and temporal RNFL than controls (Feng et al., 2016). On the other hand, age, sex, migraine history and frequency, and intraocular pressure do not seem to correlate with RNFL thickness (Lin et al., 2021), and migraineurs with and without white matter hyperintensities (WMH) have similar thicknesses (Tak et al., 2018). WMH are considered a marker of focal hypoperfusion and are associated with aura (Colombo et al., 2011). They are believed to arise from microvascular damage, are made of myelin and gliosis (Colombo et al., 2011), and are correspondingly associated with ischemia (Ulusoy et al., 2019).

In migraineurs with aura, average RNFL, superior hemisphere, and superior layer are decreased (Ulusoy et al., 2019). In migraineurs without aura, those with WMH had thinner RNF, a superior hemisphere, and superior layers than those without (Ulusoy et al., 2019). Additional non-perfusion-related factors may also contribute to WMH formation (Altunisik and Oren, 2021).

There are potential confounders to interpretation. While OCT angiography also detects depth-resolved motion contrast, reconstructing vessel perfusion at different layers of the retina, projection artifacts can get in the way of examining the layers underneath the topmost RNFL (McKendrick and Nguyen, 2022). Between studies, the retinal depths segmented for OCTA output varied, making comparison difficult (McKendrick and Nguyen, 2022), and the different layers of the eye are vulnerable to different extents to confounding by the same artifact (McKendrick and Nguyen, 2022).

### Ganglion cell layer (GCL)

Nineteen studies showed ganglion cell complex thinning in migraineurs (Güler et al., 2020). Compared to controls, both aura and non-aura migraineurs had thinner GCL (Ekinci et al., 2014; Reggio et al., 2017; Abdellatif and Fouad, 2018; Altunisik and Oren, 2021; Yurtoğulları et al., 2021), where chronicity and severity yielded greater GCL reduction (Abdellatif and Fouad, 2018). The presence of WMH did not influence this effect (Altunisik and Oren, 2021). Yet again, aura and chronic migraineurs seem most affected. Aura migraineurs had thinner GCL than non-aura migraineurs (Ascaso et al., 2017; Labib et al., 2020) and controls (Ekinci et al., 2014; Kanar et al., 2021). The corresponding reduced perfusion could explain this, especially given that migraineurs without aura do not seem to have interictal choroidal hypoperfusion. Chronic migraineurs had thinner ganglion cell layer (GCL) and complex (GCC) than controls (Uludag et al., 2014; Gunes et al., 2018; Raga-Martínez et al., 2022) and episodic migraineurs (Reggio et al., 2017). Migraineurs with more than four attacks a month had thinner GCL than controls (Tunç et al., 2017). The recurrent perfusion fluctuations and transneuronal retrograde degenerations in the primary visual cortex may cause chronic retinal damage in migraineurs, leading to ganglion cell loss (Martinez et al., 2008). GCL thinning could be a more accurate biomarker of axonal damage than RNFL (Ascaso et al., 2017). If the GCL is thinner but the RNFL is not, this may indicate fewer or shorter migraines (Gunes et al., 2018).

### Optic nerve

**Figure 3:**
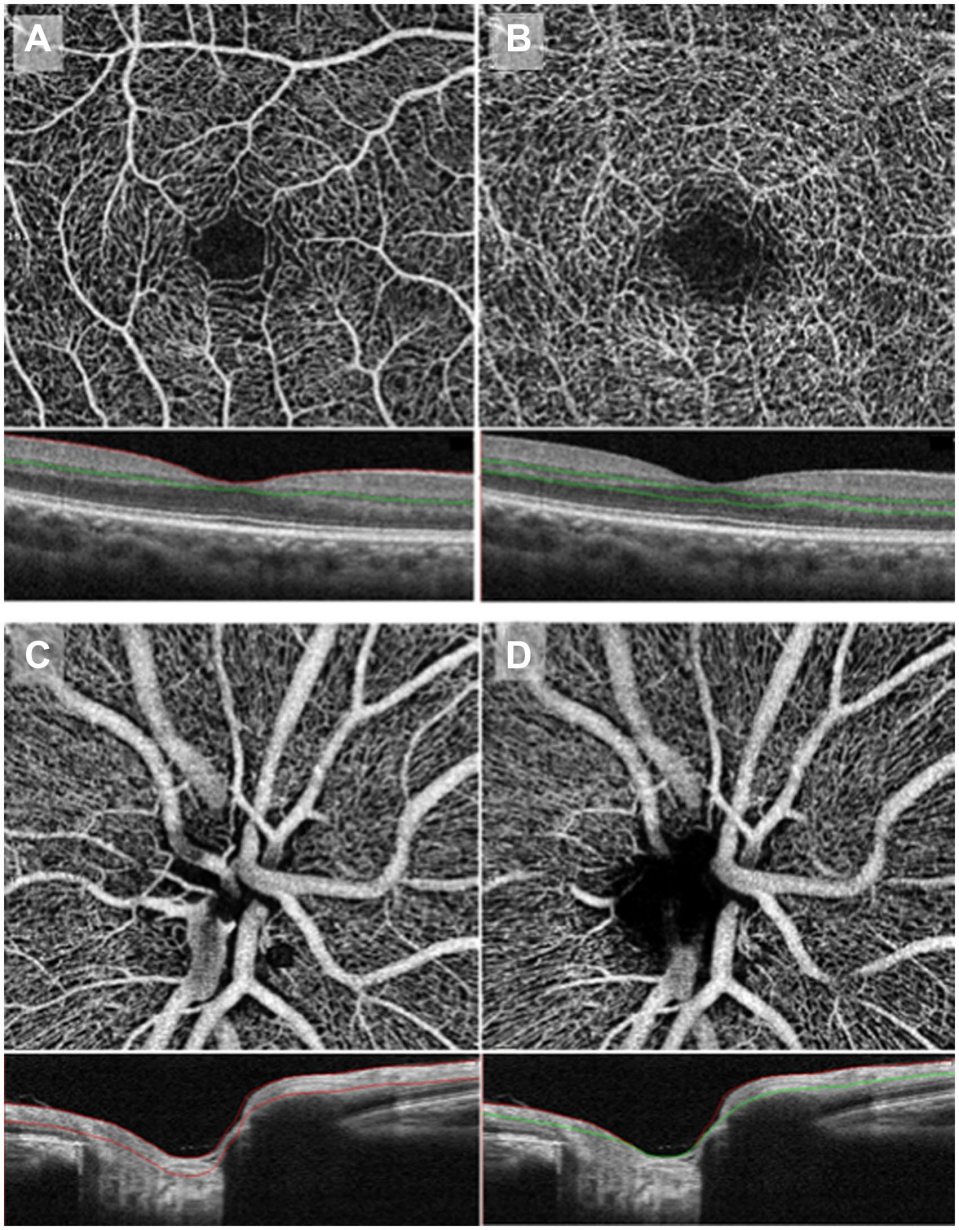
OCTA imaging of macular vessel densities in the top two panels, taken at the superficial capillary plexus **(A)** and deep capillary plexus **(B)**. OCTA imaging of the optic nerve area in the bottom two panels, taken at the superficial RPC **(C)** and deep RPC **(D)**. RPC: radial peripapillary capillary. Copied from Chang et al. (2017) under the terms of CC BY-NC-ND 4.0.

OCTA has also been able to detect optic nerve area differences between migraineurs and controls. RNFL thickness measurement has been used to monitor optic nerve diseases as an indirect measure of retinal ganglion cell loss and is not specific to migraine (Feng et al., 2016; Ascaso et al., 2017). Figures 3C,D shows an example of the optic nerve area imaged via OCTA (Chang et al., 2017).

Migraineurs, especially those with aura, tend to have optic nerve head damage (Liu et al., 2023). Migraineurs had smaller optic and neuroretinal rim disc areas, a lower cup-disc ratio at the optic nerve head, greater disc area, and larger cup volumes than controls (Khosravi et al., 2018; Yener and Yılmaz, 2020). Both aura (more so those with WMH than those without) and non-aura migraineurs had reduced whole optic disc VD compared to controls (Ulusoy et al., 2019). Different peripapillary areas of the left and right eyes may have RNFL thinning in migraineurs compared to controls (Altunisik and Oren, 2021). In some studies, investigators found no differences between aura and non-aura migraineurs (Altunisik and Oren, 2021; Kanar et al., 2021; Yurtoğulları et al., 2021).

Some areas of the RNFL may be more vulnerable than others, as seen via both depth and en-face imaging. For example, migraineurs have less superior peripapillary vessel density and marginally thinner superior RNFL than controls, suggesting that the superior retinal region may be more vulnerable to hypoperfusion (Colak et al., 2016; Sirakaya et al., 2020). The peripapillary RNFL has many unmyelinated ganglion cell axons, which require more energy to maintain, so it is more vulnerable to hypoxic damage (Wang et al., 2003). Liu et al. examined 16 studies on episodic migraineurs in their systemic review and meta-analysis, finding that aura migraineurs had exceedingly thinned peripapillary RNFL in most areas (Liu et al., 2023). Some studies reported mean peripapillary RNFL thinning in migraineurs, whereas others only in a specific quadrant (Ascaso et al., 2017). Feng’s analyzed studies showed lower mean peripapillary RNFL in migraineurs than controls (Feng et al., 2016). Peripapillary VD is lower in migraineurs and chronic hypoperfusion, which eventually leads to RNFL thinning/atrophy (Pang et al., 2023). In a meta-analysis, average peripapillary RNFL thickness was lower in migraineurs with and without aura than in controls, possibly due to vascular dysregulation and focal cerebral ischemia (Liu et al., 2023). There seems to be peripapillary RNFL thinning in all quadrants, with superior and inferior being more susceptible (which may be associated with higher ganglion axon vulnerabilities) than temporal and nasal quadrants (Liu et al., 2023), corresponding with fundus hypoperfusion occurring mostly there (Gunes et al., 2016). Migraineurs with WMH had thinner RNFL than controls (Yu et al., 2022), whereas those without WMH did not (Simsek, 2017) or had thicker RNFL than controls interictally (Yu et al., 2022). RNFL is more susceptible to damage due to greater retinal axon vulnerability than other optic areas (Gunes et al., 2016). Ictal vasospasm involving retrobulbar (ophthalmic, posterior ciliary, and central retinal) arteries may culminate in optic nerve head hypoperfusion and thus necrosis of retinal ganglion cells (Kirbas et al., 2013; Feng et al., 2016).

Again, aura migraineurs seem to have more exaggerated differences than non-aura migraineurs with controls. At the optic nerve head, aura migraineurs had reduced VD (OCTA) (Chang et al., 2017; Karahan et al., 2021) and RNFL thickness (OCT) (Sim et al., 2023) and larger optic disc rim (OCTA) (Hamurcu et al., 2021) than non-aura migraineurs and controls, with some studies finding no difference between non-aura migraineurs and controls (OCTA) (Chang et al., 2017) and some finding there was a difference between the latter two groups (also OCTA) (Güler et al., 2020). The lower the VD was, the higher the migraine frequency and severity (He et al., 2022). Decreased superior peripapillary VD and a tendency toward decreased superior peripapillary RNFL thickness in migraineurs with aura (Chang et al., 2017) suggest peripapillary hypoperfusion and that the optic nerve head may be more susceptible to damage in local hypoperfusion (McKendrick and Nguyen, 2022). Decreased VD at the optic nerve could lead to peripapillary hypoperfusion (McKendrick and Nguyen, 2022). Optic nerve injury may be caused by vascular disturbances and focal ischemia (Yülek et al., 2015), which is supported by Kara et al.’s finding of reduced perfusion in the central retinal and posterior ciliary arteries of migraineurs compared to controls (Kara et al., 2003). If the retina or optic nerve head perfusion is compromised, ganglion cell damage may ensue (Martinez et al., 2008). Migraine chronicity is characterized by ictal recurrent vasospasms and focal ischemia, which may explain optic nerve damage and peripapillary RNFL thinning (Reggio et al., 2017).

### Macula

Migraineurs had reduced macular retinal vessel and perfusion densities and thinner maculae than controls (Acer et al., 2016; Ulusoy et al., 2019; Taşlı and Ersoy, 2020; Panicker et al., 2021; Yurtoğulları et al., 2021), especially those with more than four attacks a month (Tunç et al., 2017)—the more this effect was, the higher the migraine frequency and severity (He et al., 2022). However, in some studies, investigators found similar macular thicknesses between all groups (Kirbas et al., 2013; Demircan et al., 2015; Nalcacioglu et al., 2017; Hamurcu et al., 2021). These contradictory findings could be a result of the former group of studies having slightly higher participant numbers and/or the latter group including a study with children, where we do not yet expect such differential results due to shorter migraine history. Figures 3A,B shows an example of the macular vessel densities imaged via OCTA.

Retinal ganglion cells predominate in the macula, so macular thinning is indicative of ganglion cell loss (comprised of cell bodies) and RNFL (comprised of axons) loss (Keller et al., 2014; Gupta et al., 2016; Gunes et al., 2018). In one study, investigators found no difference between those with and without WMH (Taşlı and Ersoy, 2020). Although deep foveal VD tended to be lower in migraineurs than controls, this was not significant—but this may be because of projection artifacts (Campbell et al., 2017) and the small number of studies analyzed (Pang et al., 2023). Chronic migraineurs had thinner macular RNFL and macular thickness than controls (Raga-Martínez et al., 2022).

The changes in aura migraineurs were more severe. Aura migraineurs had thinner maculae and lower VD than non-aura migraineurs and controls (Cankaya and Tecellioglu, 2016; Chang et al., 2017; Ao et al., 2019; Ulusoy et al., 2019; Karahan et al., 2021; Liu et al., 2023). In the deep capillary plexus, the VD in aura migraineurs was lower in the parafovea than in controls (Ke et al., 2022), but the measurement of the parafoveal ring differs from study to study (Ke et al., 2022). Aura migraineurs had decreased deep foveal VD ipsilateral to headache during prodromal aura, improving 3 hours post-aura (Kızıltunç and Atilla, 2020). In one study, investigators found that aura migraineurs had right-eye macular hypoperfusion during the right aura but left hemicranial pain, resolving within a week after resolution of pain and aura (González-Martín-Moro et al., 2023). Aura migraineurs had thinner maculae after 6 months post-attack (Cunha et al., 2008). Interestingly, the macular deep capillary density may be lower in all regions but the fovea in aura migraineurs (possibly due to retinal ischemia) (Liu et al., 2023).

Aura migraineurs with brain WMH had lower deeper foveal VD, thinner maculae, superior hemisphere densities, and an increased FAZ area than those without WMH (Tunç et al., 2017; Ulusoy et al., 2019). Hence, retinal VD may also be related to brain WMH (Ulusoy et al., 2019), which have been visualized in migraineurs using MRI (Seneviratne et al., 2013). In migraineurs without aura, those with WMH had lower deeper foveal and superior hemisphere VD (Ulusoy et al., 2019). However, in another study, non-aura migraineurs had lower superficial and deep macular vessel densities than controls, whether they had accompanying WMH or not (Taşlı and Ersoy, 2020), suggesting that neither WMH nor aura are sensitive markers of retinal hypoxic threat.

### Foveal avascular zone (FAZ)

**Figure 4:**
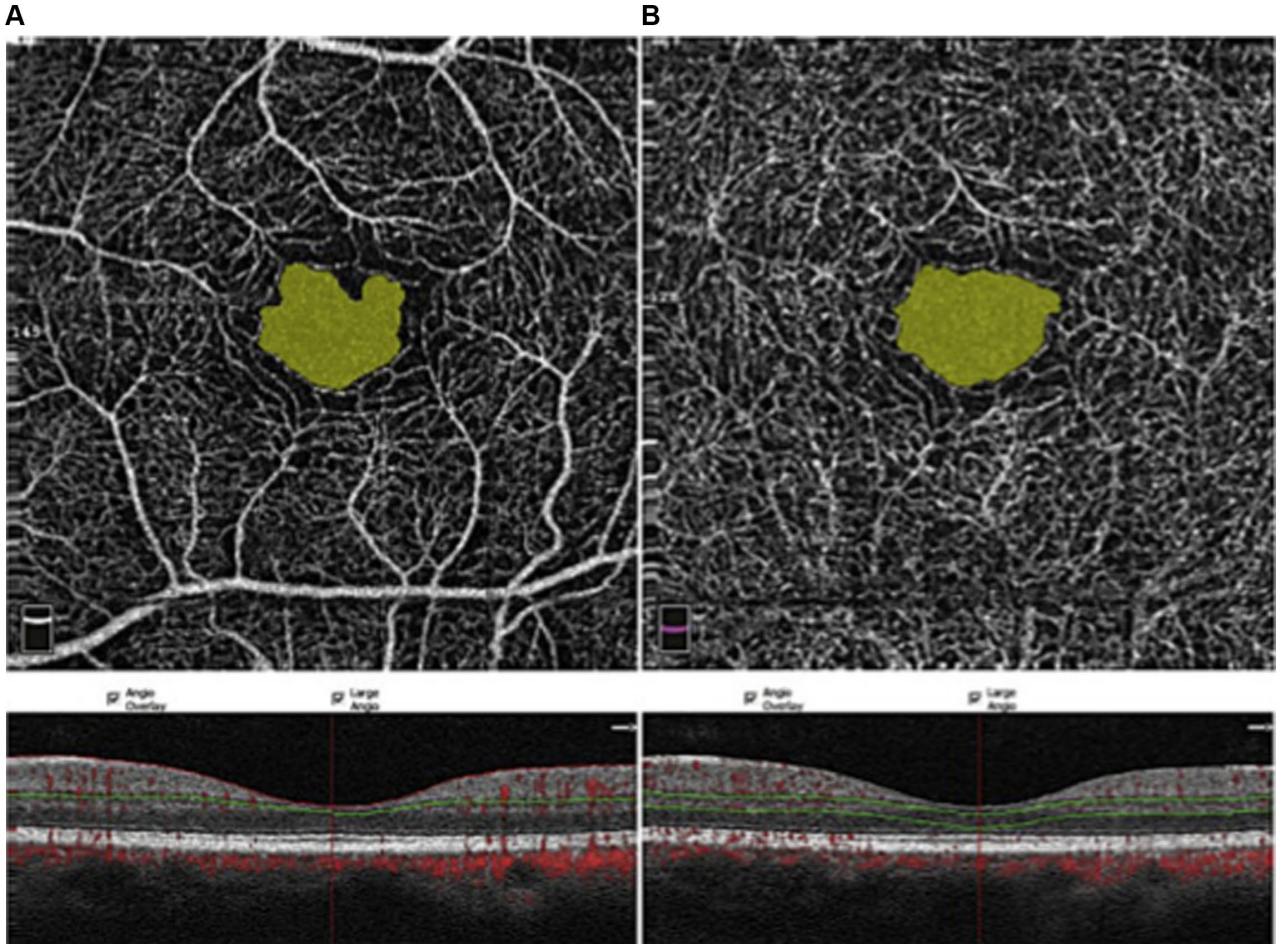
OCTA imaging of the FAZ, taken at the superficial capillary plexus **(A)** and the deep capillary plexus **(B)** (Ghassemi et al., 2017). Red dots: blood flow signals. Copied from Ghassemi et al. (2017) under the terms of CC BY-NC-ND 4.0.

The foveal avascular zone is the center of the macula. The lack of blood vessels here serves to reduce light scattering and provide maximal optical quality at the point of ocular fixation on an object. Figure 4 shows an example of the central FAZ imaged via OCTA (Ghassemi et al., 2017).

Pang’s meta-analysis of nine studies shows that migraineurs had a larger FAZ area than controls (Pang et al., 2023). Ke et al. (2022) examined nine studies, where they found that the FAZ area of aura migraineurs was greater than controls after correcting for publication bias (Ke et al., 2022). We found seven studies that looked at FAZ parameters (area and/or perimeter/circumference). They showed that the FAZ area and its perimeter were larger in migraineurs compared to controls (Chang et al., 2017; You et al., 2019; Taşlı and Ersoy, 2020; Liu et al., 2023), and most studies’ investigators found that aura migraineurs had increased FAZ area and perimeters compared to non-aura migraineurs and controls (Chang et al., 2017; You et al., 2019; Hamamci et al., 2021; Hamurcu et al., 2021; Ke et al., 2022). One study’s investigators also found that most of their aura migraineurs had deep-plexus FAZ enlargement (Karahan et al., 2021).

These observations may indicate an association between aura and ischemia (Ke et al., 2022). The larger FAZ may indicate permanent changes due to the recurrent retinal capillary ischemia causing chronic capillary ischemia or remodeling (McKendrick and Nguyen, 2022; Pang et al., 2023). FAZ area and circumference/perimeter may be greater in both aura and non-aura migraineurs, to which retinal ischemia from vasospasms may contribute (Liu et al., 2023). Recurrent attacks are associated with intracranial and intraocular vessel spasms, which result in both acute and chronic capillary and blood flow changes in the retina and trigger the FAZ enlargement via vessel death (Liu et al., 2023). In one study checking for ocular alterations and clinical differences between non-aura migraineurs and controls, investigators found no difference in FAZ size or VD, whether they had accompanying WMH or not (Taşlı and Ersoy, 2020). This suggests that neither WMH nor aura are necessarily sensitive markers of hypoxic threat. The FAZ size increases with subjective headache pain and disability scores, just as superficial and deep macular VD decrease with age and these subjective scores (Taşlı and Ersoy, 2020; Özçift et al., 2021). Women may already have larger superficial and deep FAZ than men, as seen in non-migraine volunteers (Ghassemi et al., 2017). Therefore, investigators ought to control for similar age and sex proportions between the groups tested.

## Choroid

**Figure 5:**
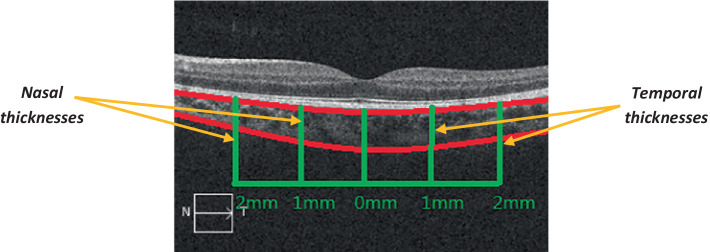
OCT cross-sectional imaging of the choroid (Li et al., 2016). The choroid is outlined in red; the green vertical lines represent its nasal and temporal thicknesses; the green horizontal lines represent the measured distances (from the fovea), to the right and the left. N: nasal; T: temporal. Adapted from Li et al. (2016) under the terms of CC BY 4.0.

OCT can also be used to measure the width of the choroid (Ascaso et al., 2017). Figure 5 shows an example of choroidal thickness measured via OCT (Li et al., 2016).

Choroidal thickness could be another indicator of hypoperfusion associated with migraine. This is because the choroid receives 95% of oxygenated ocular blood flow, so changes in choroidal structure are likely to reflect choroidal and ocular blood flow (Alm and Bill, 1970; Cioffi et al., 2003; Karalezli et al., 2015). As up to 90% of the ophthalmic artery blood supply goes to the choroid, which then perfuses the outer retina and regulates foveal heat retention, blood supply disturbances here can cause ganglion cell necrosis (Hayreh, 1975). Indeed, in a previous study, investigators showed that increased blood flow into the choroid leads to its increased thickness (Fitzgerald et al., 2002). Choroidal hypoperfusion can lead to focal ischemic damage in the optic disc (Flammer et al., 2001). Ocular pulse amplitude is an indirect indicator of choroidal perfusion, reflecting intraocular pressure fluctuation from systole to diastole, which appears to be similar in migraineurs without aura and controls (Acer et al., 2016). The greater the migraine duration and frequency, the greater the increase in subfoveal choroidal thickness in migraineurs compared to controls (Gouravani et al., 2023). Migraineurs may have thinner choroids because of reduced perfusion of the posterior ciliary artery and central retinal vessels (Tuncer et al., 2014). Reduced choroidal perfusion (with subsequent thinning) may cause ischemic damage even in the retina, such that retinal pigment epithelium, photoreceptor, and ganglion cells malfunction and die—more so in migraineurs with aura than those without (Ascaso et al., 2017).

We found 26 studies assessing the choroid in migraineurs. In general, migraineurs have [often asymmetrically between eyes (Khosravi et al., 2018)] thinner choroids than controls (Demircan et al., 2015; Zengin et al., 2015; Karaca et al., 2016; Ergiyit, 2017; Reggio et al., 2017; Khosravi et al., 2018), especially those with aura (Ekinci et al., 2014; Karaca et al., 2016; Ao et al., 2019; Kanar et al., 2021; Gouravani et al., 2023; Torun et al., 2023). This may be more pronounced in the non-central choroid (Abdellatif and Fouad, 2018). We wonder whether a self-regulatory mechanism protects the central choroid, whose function it is to supply blood to the outer retina. Gouravani et al. cross-sectionally checked macular choroids and found that migraineurs, both with and without aura, had thinner subfoveal choroids than controls, with aura migraineurs having them even thinner than non-aura migraineurs, although they only tested 10 studies. Thinner choroids in aura, compared to non-aura, migraineurs might be due to greater hypoxic damage in those with aura (Gouravani et al., 2023). During attack-free periods (seven continuous days without an attack), investigators find that choroidal thickness is decreased due to hypoperfusion. For example, in studies using OCT, investigators found that headache-side choroids were thinner in migraineurs with ≥5 migraine attacks per month, especially during attack-free periods; however, this was assessed at the same time of day to avoid diurnal variation between measurements as opposed to the same phase of the migraine cycle (Karalezli et al., 2015; Karaca et al., 2016; Unlu et al., 2017).

These results were especially associated with migraine history (Abdellatif and Fouad, 2018; Altunisik and Oren, 2021; Özçift et al., 2021), which supports the hypothesis that recurrent transient vasospasms of migraine attacks (especially with aura and trigemino-vascular system stimulation) may result in choroidal and retinal thinning (Karalezli et al., 2015; Zengin et al., 2015; Karaca et al., 2016; McKendrick and Nguyen, 2022; Gouravani et al., 2023) through posterior ciliary artery hypoperfusion (McKendrick and Nguyen, 2022). Chronic migraineurs (especially those with aura) had thinner choroids ipsilaterally to headache than controls (Colak et al., 2016; Unlu et al., 2017). In fact, where there is monocular transient loss of vision, the choroidal circulation is implicated (O’Sullivan et al., 1992). Interictally, there may also be predominating vasoconstriction in the peripheral circulation in migraine patients (detected through fingertip photoplethysmography) (Komatsu et al., 2003).

Choroidal thickness appears to increase during a migraine attack but decreases between attacks (Sorkhabi et al., 2013; Karalezli et al., 2015; Gunes et al., 2018). In one study, 46 ictal migraineurs had thicker central and peripheral choroids than controls, perhaps due to rebound vasodilation (Karalezli et al., 2015). Another study found that migraineurs ictally had thicker choroids in both eyes than controls, possibly from rebound vasodilation (Altunisik and Oren, 2021). Using OCT, investigators found that during a migraine attack, choroidal thickness was greater on the headache side with unilateral headaches (Dadaci et al., 2014). With bilateral headaches, foveal choroids increased more in the left than right eyes during migraines (Dadaci et al., 2014). The investigators attributed this phenomenon possibly to neurogenic inflammation triggered by cortical spreading depression, reflex autonomic activity due to trigemino-vascular activation (which is known to occur in migraines), or indeed altered ocular circulation (Dadaci et al., 2014). McKendrick et al. proposed that it may be due to acute vascular variations in the aftermath of the migraine attack (McKendrick and Nguyen, 2022). Considering that migraine is associated with decreased perfusion (De Simone et al., 2022), one would expect choroidal thickness to decrease during attacks. Instead, given that the choroid provides blood supply to the outer retina and sometimes thickens during an attack, it appears that the overall ocular vascular perfusion is increased interictally. We hypothesize that this acute thickening of the choroid during a migraine attack could be a cause or effect of retinal thinning. As a result, if the brain is underperfused, the choroid thickening may be induced autonomically to compensate, but that may result in an adverse effect on the vascular tone in the retina, leading to retinal thinning. As an effect, the choroid may attempt to compensate for retinal thinning by increasing its vascular tone to supply the retina. Similar to within the brain, this may instead cause further constriction of the retinal capillaries (to maintain the blood perfusion gradient within the eye), exacerbating the problem.

Counterintuitively, in three studies, investigators found choroids thinner ictally than interictally (Zengin et al., 2015) and migraineur choroids thicker than control choroids interictally (Dervisogullari et al., 2015; Altunisik and Oren, 2021; Temel et al., 2021; Çam and Arikan, 2022). In one study, investigators found chronic migraineurs have thicker choroids than controls (tested both interictally and ictally), especially during migraines (Gunes et al., 2018). These inconsistencies could be due to different severities and perfusion effects (Ascaso et al., 2017), or we think perhaps compensatory vascular changes to reduced focal blood flows surrounding attacks. Inferences were hard to make because studies rarely examined migraineurs ictally, understandably due to the difficulty in managing patients in pain.

These conflicting results may not be surprising, as the choroid is the most vascular layer of the eye and thus most sensitive to any changes in blood circulation (Yülek et al., 2015). These include acute, chronic, internal, and external changes, such as caffeine, smoking, medication, age, systemic disease, nicotine, systemic circulation issues, light exposure, diurnal rhythm, and sex (Parver, 1991; Lee et al., 2014; Allais et al., 2020; Gouravani et al., 2023), so all these factors need to be controlled in future studies. For example, menstrual/hormonal changes affect choroidal vascularity (Tan et al., 2016). While non-aura migraineurs have thinner choroids than controls, this difference becomes smaller with increasing age (Gouravani et al., 2023). Choroidal thinning especially occurs in those with chronic migraine, but some studies may be compromised in their ability to detect posterior choroidal changes due to device limitations (Ekinci et al., 2014; Reggio et al., 2017), with those using enhanced depth imaging (EDI) OCT possibly being more reliable due to better distinguishing of posterior structures of the eye. EDI OCT allows screening of 10 retinal layers and the choroid at 2 μm resolution using spectral-domain technology (Gouravani et al., 2023). In addition, a study showed that at distances of 500, 1,000, and 1,500 μm from the fovea, there were no differences in choroidal thicknesses between migraineurs and controls interictally (Gouravani et al., 2023), while others showed no difference in thickness or vascularity index between chronic migraineurs and controls (Nalcacioglu et al., 2017; Güler et al., 2020; Kanar et al., 2021; Sezer et al., 2023) (although investigators often did not specify whether they assessed interictally or ictally). For instance, some investigators found migraineurs have thicker choroids than controls, but they admit their migraineurs may have had attacks during imaging (Çam and Arikan, 2022).

## Discussion

Migraine is the world’s leading cause of disability in terms of productivity loss, given its prevalence in young working people (Feigin et al., 2019). Therefore, it is imperative for affordable, non-invasive, and efficient instruments to be developed to monitor this widespread disorder. OCT could not only be used as a diagnostic biomarker for migraine but also to track its therapy progress (Ascaso et al., 2017). We searched several databases and found papers using this technology to examine the differences between migraineurs and controls. Out of 28 studies with episodic migraineurs, 21 included those with aura and 25 included those without. Out of 19 studies with chronic migraineurs, 14 included those with aura and 14 included those without. Out of the 21 studies with episodic migraineurs with aura, 19 were cross-sectional and 2 were longitudinal. Out of the 25 studies with episodic migraineurs without aura, all 25 were cross-sectional. Out of the 14 studies with chronic migraineurs with aura, all 14 were cross-sectional. Out of the 14 studies with chronic migraineurs without aura, all 14 were cross-sectional. Therefore, it would be interesting to see more longitudinal OCT/OCTA studies with migraineurs, such as in instances where a vasoactive therapy is investigated and its effects measured before vs. after treatment (Supplementary Figure S1).

RNFL, macular, and choroidal changes have been examined as biomarker signs for the symptoms of migraine in the last few years (Ascaso et al., 2017). Within the macula, the FAZ was given particular attention. We found that studies generally show that migraineurs have thinner RNFL, GCL, and maculae, reduced optic nerve and VD parameters, and larger FAZ and their perimeters than controls. For example, in the retina, a study showed that migraineurs had lower superficial and deep macular, superficial foveal, deep parafoveal, and peripapillary VD than controls (Güler et al., 2020; Pang et al., 2023). These findings can indicate retrograde trans-synaptic neuronal degeneration of the retinal ganglion cells from the resultant ischemia in the posterior visual pathway, which can be seen as a sign in OCT scans (Ascaso et al., 2017). It may be worth investigating whether there are different hypoperfusion patterns across the cerebral cortex for the different types of vascular headache. The RNFL thinning could mostly be due to many migraine attacks chronically causing optic nerve hypoperfusion (Sirakaya et al., 2020). As the macular deep capillary density appears to have lower perfusion density in all regions but the fovea in aura migraineurs (Liu et al., 2023), we wonder whether a self-regulatory mechanism protects the fovea (as a center of retinal function) from the worst of the hypoperfusion. This calls for further investigation.

Throughout this study, there was a recurring theme of aura and chronic migraineurs having more severe versions of the same results in the various OCT and OCTA parameters than non-aura and episodic migraineurs (Supplementary Table S2). The migraine severity/duration/history/impact were also positively correlated with the severity of these findings. Interestingly, the lower superficial foveal VD and larger FAZ were detected only in females (Chang et al., 2017), corresponding with the observation that women have a higher ischemic risk (Gryglas and Smigiel, 2017). OCT and OCTA can be used to detect significant differences between patients with and without aura, potentially helping with diagnosis and therapy. Migraineurs with aura could have a higher cerebrovascular risk (Sacco et al., 2012), corresponding to a higher ischemic risk in the retina and choroid than controls and those without aura (Kanar et al., 2021), perhaps due to endothelial/smooth-muscle dysfunction and hypercoagulability (Larrosa-Campo et al., 2012). The posterior cerebral hemispheres are similarly less perfused ictally in aura migraineurs than non-aura migraineurs (Romozzi et al., 2023). Repetitive migraine attacks involving transient vasoconstrictions could ultimately result in permanent retinal and general cerebral damage (Feng et al., 2016), especially through hypoperfusion of the optic nerve, retina, and choroid through cerebral and retrobulbar vessels and retinal and ciliary arteries repetitively constricting over time in chronic migraineurs (Ascaso et al., 2017). In fact, a literature review of chronic migraine and OCT imaging admitted that brain hypoperfusion may be implicated in the pathophysiology of migraine, indicating that vascular changes detected may therefore be used as a biomarker for migraine in general (Ascaso et al., 2017).

What happens during a single migraine attack? It has been shown that cranial vasodilation is not a prerequisite, nor enough, for migraine onset (Charles, 2013); in fact, it may even be a result of the headache instead (Amin et al., 2013). Calcitonin gene-related peptide drives meningeal vasodilation, inducing transient hyperperfusion (cortical spreading depression), then a longer hypoperfusion or spreading ischemia; coupled with increased energy demand, the ischemia intensifies (Pang et al., 2023). Since ischemia can be detected in between attacks (Chang et al., 2017), this suggests a chronic vascular indicator (systemic vasoconstriction) that we can use to diagnose migraineurs (especially those with aura) even when they are not having attacks (Komatsu et al., 2003; Chang et al., 2017). Ictal changes in optic nerve and RNFL perfusion could lead to hypoxic injury and death of retinal ganglion cells (Osborne et al., 2001), possibly explaining aura and retinal vascular disease in migraineurs (Lin et al., 2021). Cortical spreading depression is said to initiate aura and can induce hyperperfusion to hypoperfusion transiently in the cortex (Liu et al., 2023). Cortical spreading depression is associated with hypoxemic injury, inflammatory activation, and a neurovascular mismatch in energy supply and demand (Lai and Dilli, 2020). Decreased VD, which resolves after 3 hours post-aura (Kızıltunç and Atilla, 2020), could be from transient vasospasm (McKendrick and Nguyen, 2022). The reduced occipital blood flow reported in some studies (Lauritzen et al., 1983; Denuelle et al., 2008), coupled with the increased neurological activity in the area (Aurora et al., 1999; Janis et al., 2010; Martín et al., 2011), may explain visual auras, which comprise 98–99% of migraine-related auras (Eriksen et al., 2004; Viana et al., 2017). This is thought to be attributed to transient cerebral vasospasm occurring around the onset of pain, which is also considered a risk factor for optic ischemic neuropathy (Ascaso et al., 2017). Visual auras from occipital cortex hypoperfusion are more common than from retinal/choroidal hypoperfusion (Ascaso et al., 2017). Migraineurs are more prone to retinal microvascular disorders (decreased VD and increased FAZ) (Pang et al., 2023).

Certain retinal layers or regions may be more vulnerable than others to the effects of hypoperfusion. This is particularly evident in the RNFL results above, perhaps due to the studies more extensively covering that layer of the eye. In addition, one study mentioned that the central macula may be more vulnerable to inflammation and hypoxic/ischemic insult than the optic nerve head (Kurtul et al., 2022). Several studies suggest that perfusion is reduced through the central retinal and posterior ciliary arteries, both ictally and interictally (Kara et al., 2003; Kirbas et al., 2013; Feng et al., 2016; Gouravani et al., 2023), culminating in optic nerve head hypoperfusion, ganglionic retinal necrosis, and choroidal vascular insufficiency (Tuncer et al., 2014; McKendrick and Nguyen, 2022). Reduced choroidal perfusion (with subsequent thinning) may cause ischemic damage in the outer retina (since the inner retina is supplied by the central retinal artery), such that the retinal pigment epithelium and photoreceptor cells malfunction and die—more so in migraineurs with aura than those without (Ascaso et al., 2017).

Other brain areas can also be affected. In previous studies, investigators have found that during a migraine attack, even without aura, there is regional hypoperfusion in the occipital areas of the brain (Woods et al., 1994; Denuelle et al., 2008). Where there is unilateral headache (with or without aura), the hypoperfusion appears to occur only on the affected (with or without aura) side (Olesen et al., 1990; Killer et al., 2003). During aura, cortical hypoperfusion could also occur in a more generalized pattern (O’brien, 1971), although in migraine attacks themselves, hypoperfusion of the optic nerve and retina has been implicated as discussed above (Martinez et al., 2008; Reggio et al., 2017). It is during those with aura that there is a greater ischemic risk (Sacco et al., 2012). Cortical thickness is related to retinal damage and higher vessel resistance (Gouravani et al., 2023). Ictally, vasospasm and compromised blood flow usually occur in one hemisphere, although other parts may also be affected, according to a case report (Killer et al., 2003). In one study, investigators found migraineurs had thicker irises than controls, which they attributed to pupillary dynamics in response to photophobia, although they did not specify how many of their migraineurs had photophobia (Çam and Arikan, 2022). OCTA studies in migraineur children may show more ocular areas affected because migraine may affect children more severely (more intense posterior retinal trigemino-vascular events), and this may be from a stronger family migraine history (hence early onset) (Yener and Yılmaz, 2020).

These changes in vascular tone can also involve the peripheral circulation, not just the cerebral (McKendrick and Nguyen, 2022). Interictally, migraineurs have been shown to have vasoconstriction in the fingertip and dermal capillary networks, as well as abnormal circulation, arteriolar resistance, and decreased vessel caliber in the retina (McKendrick and Nguyen, 2022). Interictally, migraineurs have poor perfusion and increased capillary resistance (Pang et al., 2023). Vasospasms may indicate generalized/systemic vascular dysregulation (McKendrick and Nguyen, 2022), with Flammer syndrome (a constellation of signs and symptoms due to systemic perfusion dysregulation) being a potential differential diagnosis for migraine (Konieczka et al., 2014). There are other diseases with similar OCT/OCTA findings, such as RNFL and GCL thinning in neurodegenerative diseases (Satue et al., 2016; Doustar et al., 2017; Tsokolas et al., 2020; Augustin and Atorf, 2022); therefore, these findings alone should not rule out other differential diagnoses. In fact, the vascular changes detected may reflect an ischemic mechanism underlying all of these disorders in common. An example of this is glaucoma, where vascular insufficiencies are known to be involved (McKendrick and Nguyen, 2022). Here, optic disc ischemia and optic nerve head ischemia are also present, which even increases glaucoma risk (McKendrick and Nguyen, 2022). In addition, neovascularization leads to obvious changes in both OCT and OCTA. In the case of a patient undergoing active retinal neovascularization, the changes in OCT and OCTA would confound changes to both images due to systemic conditions. Hence, investigators should assess changes in OCT and OCTA due to systemic diseases after neovascularization has been managed medically.

Other methods can be used to find additional information, such as the following: Using doppler sonography, in another study, investigators found that it is the actual vascular resistances (central retinal and posterior ciliary arteries) that are increased in migraineurs, even when they are not having an attack (Kara et al., 2003). During those attack-free periods, migraineurs with ≥5 migraine attacks per month have a greater retinal artery diameter than controls (Unlu et al., 2017). We wonder whether this is a compensatory adaptation to chronically increased oxygen demand. Using magnetic resonance imaging (MRI) scanning, WMH can be superimposed on the OCTA images to show areas especially prone to hypoperfusion in any given patient and also self-evident on the retinal OCTA.

The studies reviewed in this article were heterogeneous in various parameters and present many confounding factors that need to be addressed to reliably compare OCT and OCTA findings across studies. For instance, while the OCT and OCTA devices are named in the methodologies, their artifact-clearing abilities are not (McKendrick and Nguyen, 2022). Device type did not affect effect sizes calculated in Gouravani et al.’s review on OCT (Gouravani et al., 2023), so we did not separate studies by device type in our own analyses. However, this may be worthwhile to do in the future. Investigators should also control for age and sex proportions between groups. Based on subgroup analyses, there may also be a need for a calibrating calculation between studies using different devices to be able to make comparisons (Gouravani et al., 2023). We observed that the studies vary in methodology (device, scanned ocular areas, including foveal diameter and statistical analyses), which makes it difficult to compare results across them. So there is also a need to standardize both measurements and analysis in future studies (Pang et al., 2023). Smaller effects, such as those from migraine history and frequency, may be camouflaged by the differences in findings arising from different methodologies alone (McKendrick and Nguyen, 2022). Some parameters are not reported [such as participant migraine history or attack frequency data (Chang et al., 2017)], while others’ findings are compromised by small sample sizes (McKendrick and Nguyen, 2022; Gouravani et al., 2023; Pang et al., 2023). In particular, the studies included participants taking vasoactive medications, so this needs to be uniformly reported and controlled in the future to avoid confounding when measuring vascular tone solely in relation to migraine pathophysiology (Lin et al., 2021). Most studies were from Turkey, and we would be interested in finding out whether the findings could be generalized to migraineurs across the world. Most are cross-sectional studies, precluding us from making causative inferences as to migraine and vascular pathology, such that we cannot tell whether the findings are due to local ischemia over time or a systemic disorder that also affects the ocular vasculature (McKendrick and Nguyen, 2022; Pang et al., 2023). In our study, we noticed that the vast majority of the case–control studies seem to be cross-sectional, with only case studies looking at migraineurs longitudinally. Hence, we cannot determine cause and effect in terms of vascular tone changes and migraine outcomes. Longitudinal studies could tell us whether cortical vascular damage occurs in migraineurs before or after their migraine history. Failing this, it would help if studies recorded these vascular measurements at similar timepoints in the migraine cycles (McKendrick and Nguyen, 2022), to be able to compare longitudinally. If evaluating measurements during migraine attacks, we would recommend that measurements be taken within 3 h of onset. This is because in a recent case study, Kızıltunç et al. took measurements of the optic disc and fovea during a visual aura where migraine pain followed on the right side (Kızıltunç and Atilla, 2020). The right eye showed diffuse narrowing of the retinal vessels, as well as decreased radial peripapillary capillary and superficial and deep foveal vessel densities (Kızıltunç and Atilla, 2020). This improved 3 hours after the visual aura (Kızıltunç and Atilla, 2020). Since the right-eye pain and headache occurred after the visual aura with vascular constriction, it is possible that eye pain in migraineurs might result from hypoperfusion of the eye (Kızıltunç and Atilla, 2020).

In summary, the retina (including the macula and its fovea) and optic nerve head can be observed through the relatively new OCT and OCTA technologies, and migraine sufferers may benefit from its use as a biomarker for diagnosis, progression, and response to therapies. OCT is useful for retinal layer thickness measurement, whereas OCTA is useful to measure vascular density in different areas of the eye. Perhaps OCT and OCTA profiles can be established in the future to determine the type of migraine according to perfusion patterns across different parts of the brain, ictally and interictally. Subsequent treatment and management plans would also be influenced by this. There are many issues still to be addressed, and we keenly look forward to continued research into the use of OCT and OCTA in migraine.

## Author contributions

DC: Data curation, Formal analysis, Investigation, Methodology, Writing – original draft, Writing – review & editing. MV: Supervision, Validation, Writing – review & editing. JC: Writing – review & editing. FC: Formal analysis, Supervision, Validation, Writing – review & editing. AL: Validation, Writing – review & editing. PD: Supervision, Validation, Writing – review & editing. RT: Supervision, Writing – review & editing. VL: Supervision, Writing – review & editing. SD: Supervision, Writing – review & editing. JM: Conceptualization, Project administration, Resources, Supervision, Validation, Writing – review & editing.
