# Supplementary material for: Using optical coherence tomography and optical coherence tomography angiography to delineate neurovascular homeostasis in migraine: a review
Source: Front Neurosci. 2024 Apr 15;18:1376282. doi: 10.3389/fnins.2024.1376282 (PMC11057254; doi:10.3389/fnins.2024.1376282)
Supplement: Supplementary file 1 [file Table_1.docx]

# Supplementary material 1

| Study | Total No. Subjects | Sex of Subjects (% Female) | Adults or Children | Cross-Sectional/ or Longitudinal | Episodic or Chronic Migraineurs | Migraineurs With (MA) or Without Aura (MO) | Ictal or Interictal Measurements | Findings |
| --- | --- | --- | --- | --- | --- | --- | --- | --- |
| Abdellatif 2018 (1) | 130 | Both (71% female) | Adults | Cross-sectional | Unspecified | Both | Interictal | Migraineurs had thinner RNFL, superior and inferior GCL and all-except-central choroid than HC  The longer the migraine history, the thinner the GCL, RNFL and especially choroid  The more severe the migraine, the thinner the GCL and RNFL |
| Acer 2016 (2) | 82 | Both (91% female) | Adults | Cross-sectional | Episodic | MO | Interictal | Migraineurs had thinner temporal and nasal superior peripapillary RNFL than HC  Migraineurs tended to have thinner GCL and maculae than HC |
| Altunisik 2021 (3) | 155 | Both (77% female) | Adults | Cross-sectional | Unspecified | Both | Both | RNFL tended to be thinner in migraineurs than HC  Peripapillary RNLF in left-eye nasal, central, nasosuperior, nasoinferior quadrants and right-eye temporoinferior quadrants, was thinner in migraineurs than HC  GCL was thinner in migraineurs than HC  Choroids were thicker in migraineurs than HC  No difference in findings whether they had WMH or not  The longer they had had migraines for, the thinner the choroid  Choroids were thinner ictally than interictally  No difference in findings between MO and MA  IPL thicknesses did not differ between groups |
| Ao 2019 (4) | 165 | NA | NA | NA | NA | NA | NA | MA had thinner nasal peripapillary RNFL, inferior inner macula layer and choroid, than HC  No difference between MO and HC  The longer the migraine history or the more frequent the attacks, the thinner the nasal peripapillary RNFL |
| Bing 2019 (5) | 94 | Both (83% female) | Adults | Cross-sectional | Unspecified | Unspecified | Interictal | Migraineurs had thinner average and superior RNFL than HC |
| Bulboacă 2020 (6) | 77 | Both (68% female) | Adults | Cross-sectional | Episodic | MO | Interictal | Migraineurs had more oxidative stress markers and less anti-oxidative molecular markers, than HC. This was especially associated with the temporal quadrant  The thinner the macular inner temporal RNFL, the less the catalase (anti-oxidant marker) |
| Burgos-Blasco 2023 (7) | 37 | Both (60% female) | Children | Cross-sectional | Episodic | Both | Interictal | MA had thinner temporal and inferior temporal RNFL than MO and HC (no difference between HC and MO)  More the migraine frequency, thinner the nasal superior RNFL |
| Çam 2022 (8) | 99 | Both (66% female) | Adults | Cross-sectional | Unspecified | Unspecified | Interictal | Migraineurs had thicker iris sphincter and dilator muscle epithelia and stroma (and total, therefore) and choroids, than HC |
| Cankaya 2016 (9) | 54 | Both (56% female) | Adults | Cross-sectional | Episodic | Both | Interictal | MA had decreased foveal thickness compared to MO and HC |
| Chang 2017 (10) | 49 | Both (55% female)  HC predominantly male, MA and MO predominantly female | Adults | Cross-sectional | Unspecified | Both | Interictal | Macula: MA had increased FAZ than controls and decreased superficial foveal density than MO  Optic nerve: MA had reduced superior peripapillary VD than MO and HC  No differences between MO and HC |
| Colak 2016 (11) | 90 | Both (81% female) | Adults | Cross-sectional | Chronic | MA | Interictal | MA had thinner superior and inferior RNFL quadrants and thinner subfoveal, temporal and nasal choroids, than HC |
| Costello 2009 (12) | 123 | Unspecified | Adults | Cross-sectional | Unspecified | Both | Interictal | Temporal RNFL quadrant was thinner in migraineurs than HC  In migraineurs, the thinner the RNFL, the more the migraine disability and frequency |
| Cunha 2008 (13) | 1 | Male | Adult | Longitudinal | Unspecified | MA | Interictal | Macula and peripapillary RNFL were thinner after 6 months post-episode, at papillomacular bundle ipsilateral to scotoma development, with mild localized RNFL loss temporal to optic disc |
| Dadaci 2014 (14) | 29 | Both (86% female) | Adults | Cross-sectional | Unspecified | Both | Both | Unilateral headaches: ictal choroidal thicknesses increased ipsilaterally from basal  Bilateral headaches: foveal ictal choroidal thickness increased, more so in left eyes, compared to basal |
| Demircan 2015 (15) | 95 | Both (89% female) | Adults | Cross-sectional | Episodic | Both | Interictal | MA and MO had thinner nasal and nasal inferior RNFL than HC  Macular: similar thicknesses between all groups  MA and MO had thinner choroids than HC |
| Demirci 2016 (16) | 150 | Both (89% female) | Adults | Cross-sectional | Both | Both | Interictal | Superior, nasal and inferior RNFL thicknesses decreased in MO and MA, compared to HC  No differences between MO and MA |
| Dereli Can 2021 (17) | 101 | Both (63% female) | Children | Cross-sectional | Unspecified | MO | Interictal | No difference between groups  The less the capillary VD and RNFL thickness, the more the migraine disability |
| Dervisogullari 2015 (18) | 59 | Both (85% female) | Adults | Cross-sectional | Unspecified | Both | Ictal | Ictally, choroid was thinner in migraineurs than HC |
| Ekinci 2014 (19) | 120 | Both (67% female) | Adults | Cross-sectional | Unspecified | Both | Interictal | MA had thinner RNFL, GCL and choroid than MO and HC  MO and especially MA had thinner choroids than HC  Migraineurs overall had thinner RNFL, GCC and choroid than HC |
| Ergiyit 2017 (20) | 72 | NA | NA | NA | NA | NA | NA | Migraineurs had thinner temporal upper, temporal lower, inferonasal and mean RNFL and choroid than HC |
| El-Shazly 2017 (21) | 90 | Both (69% female) | Adults | Longitudinal | Episodic | MA | Both | Ictally, MA had thinner RNFL than HC, thickening interictally but remaining thinner than HC |
| Gipponi 2013 (22) | 40 | Female | Adults | Cross-sectional | Episodic | Both | Interictal | Migraineurs had decreased superior quadrant thicknesses compared to HC  The thinner the RNFL, the longer the migraine history and attack and aura durations |
| González-Martín-Moro 2023 (23) | 1 | Male | Adult | Longitudinal | Unspecified | MA | Both | During left hemicranial pain and right-eye aura, there was right-eye macular hypoperfusion which resolved 2-7 days after aura and pain resolution |
| Guler 2020 (24) | 50 | Both (88% female) | Adults | Cross-sectional | Episodic | MO | Ictal | Migraineurs had less deep superior VD than HC |
| Gunes 2016 (25) | 116 | Both (84% female) | Adults | Cross-sectional | Episodic | Both | Interictal | Tendency for average and nasal thicknesses to be thinner in migraineurs than controls, more so on ipsilateral headache sides |
| Gunes 2018 (26) | 81 | Both (67% female) | Adults | Cross-sectional | Chronic | Both | Both | Choroidal thickness was increased and GCL decreased, in chronic migraineurs than HC  Choroid was thicker during than between migraines  Cornea was thicker during migraines than between or HC |
| Hamamci 2021 (27) | 90 | Both (75% female) | Adults | Cross-sectional | Episodic | Both | Interictal | MA had lower superficial and deep foveal, whole optic disc, optic disc inside, peripapillary, superior hemisphere, inferior hemisphere, superior quadrant and temporal quadrant VD and larger FAZ, than HC  The lower the VD, the higher the migraine frequency, disability and life impact |
| Hamurcu 2021 (28) | 38 | Both (89% female) | Adults | Cross-sectional | Unspecified | MA | Interictal | No thickness differences between groups  MA had larger optic disc rim area than HC  MA had larger FAZ than HC  MA tended to have lower vessel densities than HC  The longer MA had had migraines for, the lower the superior parafoveal VD tended to be |
| He 2022 (29) | 86 | Both (87% female) | Adults | Cross-sectional | Both | Both | Interictal | Migraineurs had reduced macular retinal vessel and perfusion densities than HC  MA had reduced retinal vessel density at the optic nerve head than HC  The lower the retinal vessel and perfusion densities of the macula and optic nerve head, the higher the migraine frequency and severity |
| Iyigundogdu 2018 (30) | NA | NA | NA | NA | NA | NA | NA | NA |
| Kanar 2021 (31) | 127 | Both (72% female) | Adults | Cross-sectional | Episodic | Both | Interictal | Foveal, nasal and temporal choroids were thinner in MA than MO or HC  There was no difference in choroidal thickness between MO and HC  Both MO and MA had thinner global, superior and inferior peripapillary RNFL than HC, with MO and MA having no difference  Nasal quadrant peripapillary RNFL was thinner in MA than in MO or HC  Superior and inferior macular GCL were thinner in MA and MO than in HC |
| Karaca 2016 (32) | 64 | Female (but 1 male HC, 1 male MO) | Adults | Cross-sectional | Both | Both | Interictal | MA and MO had thinner choroidal thicknesses than HC, with MA tending to be thinner than MO |
| Karahan 2021 (33) | 116 | NA | NA | Cross-sectional | NA | MA | Interictal | MA had decreased vessel densities at nasal and inferotemporal optic nerve head, inferonasal radial peripapillary capillaries and deep macular plexus, than HC  Most MA had deep FAZ enlargement |
| Karalezli 2015 (34) | 46 | Both (52% female) | Adults | Cross-sectional | Unspecified | Unspecified | Ictal | Migraineur choroids were ictally thicker than controls |
| Khosravi 2018 (35) | 60 | Both (65% female) | Adults (but youngest were less than 18 years old) | Cross-sectional | Unspecified | Both | Interictal | Migraineurs had thinner and less eye-eye symmetrical RNFL and choroid, smaller optic and neuroretinal rim disk area and lower cup-disk ratio at optic nerve head, than HC |
| Kirbas 2013 (36) | 80 | Both (63% female) | Adults | Cross-sectional | Chronic | Unspecified | Interictal | Only superior RNFL thickness was lower in chronic migraineurs than HC  No macular differences between groups |
| Kızıltunç 2020 (37) | 1 | Female | Adult | Longitudinal | Episodic | MA | Both | During aura preceding headache, diffuse retinal vessel narrowing, decreased radial peripapillary capillary density, decreased deep foveal VD. These changes occurred only ipsilaterally and improved 3 hours after aura |
| Kızıltunç 2020 (38) | 61 | Female | Adults | Cross-sectional | Unspecified | Both | Interictal | MA had less choriocapillaris blood flow than MO and HC |
| Kurtul 2022 (39) | NA | NA | Children | NA | NA | NA | NA | NA |
| Labib 2020 (40) | 60 | Both (83% female) | Adults | Cross-sectional | Chronic | Both | Interictal | Chronic migraineurs had more thinning of average, superior, inferior, nasal, temporal RNFL than HC  MA had thinner average RNFL and GCL than MO |
| Martinez 2008 (41) | 123 | Both (NA) | Adults | Cross-sectional | Unspecified | Both | Interictal | Migraineurs had thinner temporal RNFL than HC and higher migraine frequency and disability were associated with thinner RNFL |
| Midelfart 2013 (42) | NA | NA | NA | NA | NA | NA | NA | NA |
| Nalcacioglu 2017 (43) | 80 | Both (75% female) | Children | Cross-sectional | Episodic | Both | Interictal | No difference between groups |
| Oba 2023 (44) | 70 | Females | Adults | Cross-sectional | Both | MO | Interictal | Chronic MO had thinner superior temporal RNFL and lower temporal and temporal inferior circumpapillary VD than HC (indicating optic nerve damage) |
| Özçift 2021 (45) | 70 | Both (76% female) | Adults | Cross-sectional | Unspecified | Unspecified | Interictal | Longer the migraine history, thinner the choroid |
| Panicker 2021 (46) | 111 | Both (81% female) | Adults | Cross-sectional | Unspecified | Both | Interictal | Peripapillary, temporal and nasal RNFL quadrant thickness and central macular thickness, were decreased in migraineurs than HC |
| Raga-Martinez 2022 (47) | 180 | Both (86% female) | Adults | Cross-sectional | Chronic | Both | Interictal | Chronic migraineurs had thinner superior quadrant peripapillary and macular RNFL, macula, GCL than HC |
| Reggio 2017 (48) | 77 | Both (82% female) | Adults | Cross-sectional | Both | Both | Interictal | Migraineurs had decreased RNFL, GCL and choroid thicknesses than HC  Chronic migraineurs had more reduced RNFL and GCL than episodic migraineurs |
| Romozzi 2023 (49) | 60 | Both (77% female) | Adults | Cross-sectional | Both | Both | Interictal | MA had larger FAZ than HC  MA had lower foveal choriocapillaris VD than MO |
| Salman 2015 (50) | 120 | Both (58% female) | Adults | Cross-sectional | Episodic | Both | Interictal | No thickness differences between migraineurs and HC |
| Sezer 2023 (51) | 72 | Both (83% female) | Adults | Cross-sectional | Chronic | Both | Interictal | No differences in choroidal thicknesses or vascularity indices between HC, MO and MA |
| Sim 2023 (52) | 75 | NA | Children | NA | NA | Both | NA | Optic disk RNFL was lower in MA than HC and MO |
| Simsek 2015 (53) | 80 | Both (73% female) | Adults | Cross-sectional | Both | Both | Interictal | No differences in RNFL thickness were found between MO, MA or HC – except nasal quadrant of right eye which was thicker in migraineurs than HC |
| Simsek 2017 (54) | 80 | Unspecified | Adults | Cross-sectional | Unspecified | Both | Interictal | Migraineurs with WMH had thinner RNFL than HC, but migraineurs without WMH did not |
| Sirakaya 2020 (55) | 97 | Both (85% female) | Adults | Cross-sectional | Unspecified | Both | Interictal | MA and MO had thinner RNFL and thinner and deeper lamina cribrosa, than HC  The longer they had migraines for, the thinner the RNFL  The thinner the lamina cribrosa, the higher the migraine disability |
| Slagle 2021 (56) | 1 | NA | NA | NA | NA | NA | NA | NA |
| Sorkhabi 2013 (57) | 90 | Both (71% female) | Adults | Cross-sectional | Chronic | Both | Interictal | Migraineurs had thinner RNFL in the nasal quadrant only, than HC and there was no difference between those with and without aura |
| Tak 2018 (58) | 120 | Both (89% female) | Adults | Cross-sectional | Unspecified | Unspecified | Interictal | Migraineurs had thinner RNFL than HC  No difference was found between migraineurs with and without WMH |
| Taşlı 2020 (59) | 109 | Both (78% female) | Adults | Cross-sectional | Episodic | MO | Interictal | MO had larger FAZ and lower superficial and deep macular VDs, than HC  No differences between MO with vs. without WMH |
| Temel 2021 (60) | 56 | Both (52% female) | Adults | Cross-sectional | Episodic | MO | Interictal | Migraineurs had thinner non-nasal RNFL and increased choroidal vascularity, than HC |
| Torun 2023 (61) | 138 | Both (93% female) | Adults | Cross-sectional | Unspecified | Both | Interictal | MO and especially MA had thinner choroids  Those with visual aura had thinner RNFL than non-visual aura |
| Tunç 2017 (62) | 120 | Both (83% female) | Adults | Cross-sectional | Unspecified | Both | Unspecified | Migraineurs had thinner average, inferior and superior RNFL than HC  MA had thinner average, inferior and superior RNFL quadrants than MO and HC; MO had thinner average and inferior RNFL than HC  Migraineurs with >4 attacks a month had thinner macular parts and GCL than HC  Migraineurs with WMH had thinner macular parts than those without |
| Uludag 2014 (63) | 40 | NA | Adults | NA | Chronic | NA | NA | RNFL and GCC were thinner in chronic migraineurs than HC |
| Ulusoy 2019 (64) | 88 | Both (72% female) | Adults | Cross-sectional | Episodic | Both | Interictal | Macula: superficial and deep retinal foveal VD reduced in MO and MA than HC  Optic nerve: whole optic disc, peripapillary, superior hemisphere, superior layer and temporal layer VD reduced in MO and MA than HC  In MA, those with WMH had reduced deeper foveal VD and superior hemisphere VD, average RNFL, superior hemisphere and superior layer than those without WMH |
| Ulusoy 2019 (65) | 98 | Both (66% female) | Adults | Cross-sectional | Episodic | Unspecified | Interictal | In temporal and medial quadrants, the thinner the RNFL, the higher the migraine severity and disability |
| Unlu 2017 (66) | 104 | Both (84% female) | Adults | Cross-sectional | Both | Both | Interictal | In chronic migraineurs but not episodic, retinal artery diameters increased and choroidal thicknesses decreased ipsilaterally to headache, compared to HC  No changes in retinal vein diameters were found between groups |
| Verroiopoulos 2016 (67) | 38 | Both (92% female) | NA | NA | NA | Both | NA | MA had superior and inferior RNFL quadrants thinner than HC  MO had superior RNFL quadrant thinner than HC |
| Yener 2019 (68) | 70 | Both (86% female) | Adults | Cross-sectional | Unspecified | Unspecified | Interictal | No differences between groups |
| Yener 2020 (69) | 50 | Both (76% female) | Children | Cross-sectional | Unspecified | MO | Interictal | MO had thicker nasal quadrant RNFL than HC  MO had thinner temporal quadrant left-eye RNFL than HC  MO had greater left-eye disc area than HC  MO had larger cup volumes than HC |
| Yu 2022 (70) | 100 | Both (78% female) | Adults | Cross-sectional | Episodic | Both | Interictal | Vestibular migraineurs with WMH had thinner RNFL than HC  Vestibular migraineurs without WMH had thicker RNFL than HC  MO and MA had similar RNFL thicknesses |
| Yülek 2015 (71) | 100 | Both (71% female) | Adults | Cross-sectional | Unspecified | Both | Interictal | Migraineurs had decreased RNFL thicknesses compared to HC  Longer the migraine history, thinner the RNFL |
| Yurtoğulları 2021 (72) | 126 | Both (79% female) | Adults | Cross-sectional | Both | Both | Interictal | MO and MA had thinner central and inner inferior macula; central macular RNFL; inner inferior and temporal, outer nasal and outer GCL, than HC |
| Zengin 2015 (73) | 84 | Both (86% female) | Adults (but lowest below 18 years old) | Cross-sectional | Unspecified | Both | Both | Migraineurs had thinner choroids than HC  Ictally, migraineurs had thinner choroids than interictally |

***Table 1.*** *OCT and OCTA studies in migraineurs shown alongside study and migraineur types, measurement timings and vessel density results. Study authors listed in alphabetical order. FAZ: foveal avascular zone; RNFL: retinal nerve fibre layer; GCL: ganglion cell layer; GCC: ganglion cell complex; IPL: inner plexiform layer; MA: migraineurs with aura; MO: migraineurs without aura; HC: healthy controls; VD: vessel density; WMH: white matter hyperintensity; NA: data not available (full text unavailable). Chronic migraine classification: 15/+ attacks/month (frequency) or 15/+ days/month (duration).*

1. Abdellatif MK, Fouad MM. Effect of duration and severity of migraine on retinal nerve fiber layer, ganglion cell layer, and choroidal thickness. European Journal of Ophthalmology. 2018;28(6):714-21.

2. Acer S, Oğuzhanoğlu A, Çetin EN, Ongun N, Pekel G, Kaşıkçı A, et al. Ocular pulse amplitude and retina nerve fiber layer thickness in migraine patients without aura. BMC ophthalmology. 2016;16(1):1-8.

3. Altunisik E, Oren B. Retinal neurovascular structural changes in optical coherence tomography and the relationship between these changes and white matter hyperintensities in patients with migraine. European Neurology. 2021;84(6):460-71.

4. Ao R, Wang R, Yang M, Wei S, Shi X, Yu S. Altered retinal nerve fiber layer thickness and choroid thickness in patients with migraine. European Neurology. 2019;80(3-4):130-7.

5. Bing MND, Adil H, Sanihah ABDH, Azrin ABHS. Evaluation of Optic Nerve Head Parameters, Retinal Nerve Fiber Layer Thickness, and Ocular Perfusion Pressure in Migraine Patients. Cureus. 2019;11(5).

6. Bulboacă AE, Stănescu IC, Bolboacă SD, Bulboacă AC, Bodizs GI, Nicula CA. Retinal nerve fiber layer thickness and oxidative stress parameters in migraine patients without aura: a pilot study. Antioxidants. 2020;9(6):494.

7. Burgos-Blasco B, Ginés-Gallego C, Carrasco-López-Brea M, de Santos-Moreno MT, Santos-Bueso E. Retinal Nerve Fiber Layer Analysis in Children With Migraine With and Without Aura Using Optical Coherence Tomography: A Case–Control Study. Journal of Pediatric Ophthalmology and Strabismus. 2023;60(3):196-202.

8. Çam M, Arikan S. Evaluation of iris epithelial and stromal thickness in patients with migraine by using optical coherence tomography. Neurology Asia. 2022;27(1).

9. Cankaya C, Tecellioglu M. Foveal thickness alterations in patients with migraine. Medical Archives. 2016;70(2):123.

10. Chang MY, Phasukkijwatana N, Garrity S, Pineles SL, Rahimi M, Sarraf D, et al. Foveal and peripapillary vascular decrement in migraine with aura demonstrated by optical coherence tomography angiography. Investigative ophthalmology & visual science. 2017;58(12):5477-84.

11. Colak HN, Kantarcı FA, Tatar MG, Eryilmaz M, Uslu H, Goker H, et al. Retinal nerve fiber layer, ganglion cell complex, and choroidal thicknesses in migraine. Arquivos brasileiros de oftalmologia. 2016;79:78-81.

12. Costello F. Retinal Nerve Fiber Layer Thickness Measurements Using Optical Coherence Tomography in Migraine Patients. Evidence-Based Ophthalmology. 2009;10(2):84-5.

13. Cunha LP, Vessani RM, Ribeiro Monteiro ML. Localized neural loss detected by macular thickness reduction using optical coherence tomography: Case report. Arquivos Brasileiros de Oftalmologia. 2008;71(5):743-6.

14. Dadaci Z, Doganay F, Acir NO, Aydin HD, Borazan M. Enhanced depth imaging optical coherence tomography of the choroid in migraine patients: implications for the association of migraine and glaucoma. British Journal of Ophthalmology. 2014;98(7):972-5.

15. Demircan S, Ataş M, Arık Yüksel S, Ulusoy MD, Yuvacı İ, Arifoğlu HB, et al. The impact of migraine on posterior ocular structures. Journal of ophthalmology. 2015;2015.

16. Demirci S, Gunes A, Demirci S, Kutluhan S, Tok L, Tok O. The effect of cigarette smoking on retinal nerve fiber layer thickness in patients with migraine. Cutaneous and ocular toxicology. 2016;35(1):21-5.

17. Dereli Can G, Can ME, Ekici A. Evaluation of retinal microvasculature and foveal avascular zone by the optical coherence tomography angiography in pediatric migraine patients. Acta Neurologica Belgica. 2021;121(6):1449-55.

18. Dervisogullari M, Totan Y, Gençler O. Choroid thickness and ocular pulse amplitude in migraine during attack. Eye. 2015;29(3):371-5.

19. Ekinci M, Ceylan E, Çağatay HH, Keleş S, Hüseyinoğlu N, Tanyıldız B, et al. Retinal nerve fibre layer, ganglion cell layer and choroid thinning in migraine with aura. BMC ophthalmology. 2014;14(1):1-6.

20. Ergiyit T. To evaluate the choroidal thickness and retinal nerve fiber layer thickness in patients with migraine | Migren Hastalarýnda Koroid Kalýnlýǧý ve Retina Sinir Lifi Kalýnlýǧýnýn Deǧerlendirilmesi. Retina-Vitreus. 2017;26(3):242-5.

21. El-Shazly AAE-F, Farweez YA, Hamdi MM, El-Sherbiny NE. Pattern Visual Evoked Potential, Pattern Electroretinogram, and Retinal Nerve Fiber Layer Thickness in Patients with Migraine during and after Aura. Current eye research. 2017;42(9):1327-32.

22. Gipponi S, Scaroni N, Venturelli E, Forbice E, Rao R, Liberini P, et al. Reduction in retinal nerve fiber layer thickness in migraine patients. Neurological Sciences. 2013;34:841-5.

23. González-Martín-Moro J, Porta Etessam J, Pilo de la Fuente B, Fuentes Vega I, Contreras I. Acute monocular oligemia in a patient with migraine with aura demonstrated using OCT-angiography: A case report. European Journal of Ophthalmology. 2023;33(3):NP52-NP5.

24. Güler Ö, Güler M, Tuğan Yıldız CB, Hakkoymaz H. Are Retinal and Peripapillary Blood Flows Affected during Migraine Attack? Neuroophthalmology. 2020;44(5):299-306.

25. Gunes A, Demirci S, Tok L, Tok O, Demirci S, Kutluhan S. Is retinal nerve fiber layer thickness change related to headache lateralization in migraine? Korean Journal of Ophthalmology. 2016;30(2):134-9.

26. Gunes A, Karadag AS, Yazgan S, Celik HU, Simsek A. Evaluation of retinal nerve fibre layer, ganglion cell layer and choroidal thickness with optical coherence tomography in migraine patients: a case‐control study. Clinical and Experimental Optometry. 2018;101(1):109-15.

27. Hamamci M, Songur MS, Bayhan SA, Bayhan HA. Is ocular vascularity affected in young migraine patients? A pilot study. Journal of Clinical Neuroscience. 2021;91:144-51.

28. Hamurcu MS, Gultekin BP, Koca S, Ece SD. Evaluation of migraine patients with optical coherence tomography angiography. International Ophthalmology. 2021;41(12):3929-33.

29. He N, Shao H, He J, Zhang X, Ye D, Lv Z. Evaluation of retinal vessel and perfusion density in migraine patients by optical coherence tomography angiography. Photodiagnosis and Photodynamic Therapy. 2022;40:103060.

30. Iyigundogdu I, Derle E, Asena L, Kural F, Kibaroglu S, Ocal R, et al. Relationship between white matter hyperintensities and retinal nerve fiber layer, choroid, and ganglion cell layer thickness in migraine patients. Cephalalgia. 2018;38(2):332-9.

31. Kanar HS, Toz HT, Penbe A. Comparison of retinal nerve fiber layer, macular ganglion cell complex and choroidal thickness in patients with migraine with and without aura by using optical coherence tomography. Photodiagnosis and Photodynamic Therapy. 2021;34:102323.

32. Karaca EE, Koçer EB, Özdek Ş, Akçam HT, Ercan MB. Choroidal thickness measurements in migraine patients during attack-free period. Neurological Sciences. 2016;37(1):81-8.

33. Karahan M, Erdem S, Ava S, Kaya A, Demirtas A, Keklikci U. Evaluation of retinal and optic nerve vasculature by optic coherence tomography angiography in Migraine with Aura. Journal Français d'Ophtalmologie. 2021;44(9):1396-402.

34. Karalezli A, Celik G, Koktekir BE, Kucukerdonmez C. Evaluation of choroidal thickness using spectral-domain optical coherence tomography in patients with migraine: a comparative study. European journal of ophthalmology. 2015;25(4):348-52.

35. Khosravi A, Shahraki K, Moghaddam A. Assessment of retinal nerve fiber layer thickness in migraine patients measured with optical coherence tomography. Int J Adv Med. 2018;5:3.

36. Kirbas S, Tufekci A, Turkyilmaz K, Kirbas A, Oner V, Durmus M. Evaluation of the retinal changes in patients with chronic migraine. Acta Neurologica Belgica. 2013;113:167-72.

37. Kızıltunç PB, Atilla H. Vascular changes with optical coherence tomography angiography during aura of migraine: A case report. European Journal of Ophthalmology. 2020:1120672119899900.

38. Kızıltunç PB, Özcan G, Özer F, Işıkay C, Atilla H. Evaluation of retinal vessel density and choriocapillaris flow in migraine patients with and without aura. Graefe's Archive for Clinical and Experimental Ophthalmology. 2020;258(11):2517-21.

39. Kurtul BE, Sipal C, Akbas Y. Assessment of the optic disc and retinal microvasculature by optical coherence tomography angiography in patients with pediatric migraine. Journal of Neuro-Ophthalmology. 2022:10.1097.

40. Labib DM, Hegazy M, Esmat SM, Ali EAH, Talaat F. Retinal nerve fiber layer and ganglion cell layer changes using optical coherence tomography in patients with chronic migraine: a case-control study. The Egyptian Journal of Neurology, Psychiatry and Neurosurgery. 2020;56:1-6.

41. Martinez A, Proupim N, Sanchez M. Retinal nerve fibre layer thickness measurements using optical coherence tomography in migraine patients. British Journal of Ophthalmology. 2008;92(8):1069-75.

42. Midelfart A, Bjurstrøm HA, Sollie SR, Midelfart E, Midelfart S. Visual field defects and retinal nerve fibre layer thickness measurements using optical coherence tomography in migraine patients. Investigative Ophthalmology & Visual Science. 2013;54(15):4380-.

43. Nalcacioglu P, Taslipinar Uzel AG, Uzel MM, Cagil N, Citak Kurt AN. Are there any changes in posterior ocular structure parameters in pediatric migraine patients? European Journal of Ophthalmology. 2017;27(4):495-501.

44. Oba T, Gulec ZEK, Cicek F, Uygunoglu U, Onder F. Retinal and peripapillary vascular density in episodic and chronic migraine cases without aura. Photodiagnosis and Photodynamic Therapy. 2023;44:103809.

45. Özçift SG, Aydın E, Eriş E. Assessment of the choroidal thickness, central macular vascular and optic disk perfusion in migraine patients with optical coherence tomography angiography. Photodiagnosis and Photodynamic Therapy. 2021;35:102397.

46. Panicker G, Kaliaperumal S, Narayan S, Mani M. Glaucoma and optical coherence tomography changes in migraine: A comparative cross-sectional study. Indian Journal of Ophthalmology. 2021;69(12):3546.

47. Raga-Martínez I, Povedano-Montero FJ, Hernández-Gallego J, López-Muñoz F. Decrease Retinal Thickness in Patients with Chronic Migraine Evaluated by Optical Coherence Tomography. Diagnostics. 2022;13(1):5.

48. Reggio E, Chisari CG, Ferrigno G, Patti F, Donzuso G, Sciacca G, et al. Migraine causes retinal and choroidal structural changes: evaluation with ocular coherence tomography. Journal of neurology. 2017;264(3):494-502.

49. Romozzi M, Cuffaro G, Rollo E, Mattei R, Marcelli S, Rizzo S, et al. Microvascular involvement in migraine: an optical coherence tomography angiography study. Journal of Neurology. 2023.

50. Salman AG, Hamid MAA, Mansour DE. Correlation of visual field defects and optical coherence tomography finding in migraine patients. Saudi Journal of Ophthalmology. 2015;29(1):76-80.

51. Sezer T, Ayaslı AAH, Meydan B. Differences in Choroidal Vascularity Index Between Migraine Patients in the Attack-free Period and Normal Individuals. Retina-Vitreus/Journal of Retina-Vitreous. 2023;32(2).

52. Sim HE, You SJ, Choi J. Analysis of Retinal Nerve Fiber Layer Thickness in Pediatric Migraine Using Optical Coherence Tomography. Journal of the Korean Ophthalmological Society. 2023;64(10):951-6.

53. Simsek IB, Aygun D, Yildiz S. Retinal nerve fibre layer thickness in migraine patients with or without aura. Neuro-Ophthalmology. 2015;39(1):17-21.

54. Simsek IB. Retinal nerve fibre layer thickness of migraine patients with or without white matter lesions. Neuro-Ophthalmology. 2017;41(1):7-11.

55. Sirakaya E, Kucuk B, Agadayi A, Yilmaz N. Evaluation of the lamina cribrosa thickness and depth in patients with migraine. International Ophthalmology. 2020;40:89-98.

56. Slagle WS, Sheets SR, Logan AB, Epps M, John VJ. Case Report: Retinal Infarction Associated with Migraine. Optometry and vision science :. 2021;98(10):1132-8.

57. Sorkhabi R, Mostafaei S, Ahoor M, Talebi M. Evaluation of retinal nerve fiber layer thickness in migraine. Iranian journal of neurology. 2013;12(2):51.

58. Tak AZA, Sengul Y, Bilak Ş. Evaluation of white matter hyperintensities and retinal fiber layer, ganglion cell layer, inner-plexiform layer, and choroidal layer in migraine patients. Neurological Sciences. 2018;39:489-96.

59. Taşlı NG, Ersoy A. Altered Macular Vasculature in Migraine Patients without Aura: Is It Associated with Ocular Vasculature and White Matter Hyperintensities? Journal of Ophthalmology. 2020;2020.

60. Temel E, Aşikgarip N, Koçak Y, Şahin C, Özcan G, Kocamiş Ö, et al. Choroidal vascularity index and retinal nerve fiber layer reflectivity in newly diagnosed migraine patients. Photodiagnosis and Photodynamic Therapy. 2021;36:102531.

61. Torun IM, Dikmen NT, Unsal MA, Sonmez M. Evaluation of the choroidal vascular index and choroidal changes in migraine subgroups. Photodiagnosis and Photodynamic Therapy. 2023;42:103348.

62. Tunç A, Güngen BD, Evliyaoğlu F, Aras YG, Tekeşin AK. Evaluation of retinal nerve fiber layer, ganglion cell layer and macular changes in patients with migraine. Acta Neurologica Belgica. 2017;117:121-9.

63. Uludag G, Ozer D, Tanyildiz B, Ceylan E, Cinici E. Assessment of Retinal Nerve Fiber Layer and Ganglion Cell Complex Thickness Using Optical Coherence Tomography in Chronic Migraine Patients. Glokom-Katarakt. 2014;9(4).

64. Ulusoy MO, Horasanlı B, Kal A. Retinal vascular density evaluation of migraine patients with and without aura and association with white matter hyperintensities. Acta Neurologica Belgica. 2019;119(3):411-7.

65. Ulusoy DM, Ulusoy EK, Duru Z. Evaluation of retinal nerve fibre layer and ganglion cell complex thickness with optical coherence tomography in migraine patients. 2019.

66. Unlu M, Sevim DG, Gultekin M, Baydemir R, Karaca C, Oner A. Changes in retinal vessel diameters in migraine patients during attack-free period. International journal of ophthalmology. 2017;10(3):439.

67. Verroiopoulos GV, Nitoda E, Ladas ID, Brouzas D, Antonakaki D, Moschos MM. Ophthalmological assessment of OCT and electrophysiological changes in migraine patients. Journal of Clinical Neurophysiology. 2016;33(5):431-42.

68. Yener AÜ, Korucu O. Quantitative analysis of the retinal nerve fiber layer, ganglion cell layer and optic disc parameters by the swept source optical coherence tomography in patients with migraine and patients with tension-type headache. Acta Neurologica Belgica. 2019;119:541-8.

69. Yener AÜ, Yılmaz D. Topographic changes measured by the swept source optical coherence tomography in retinal nerve fiber layer, optic nerve head and macula in children with migraine. Acta Neurologica Belgica. 2020;120(3):661-8.

70. Yu S, Defu L, Mengyao H, Qian C, Zhuo H, Lijun X. Retinal nerve fiber layer thickness and visual aura mechanism in patients with vestibular migraine with or without white matter lesions. Neurology Asia. 2022;27(3):709-16.

71. Yülek F, Dirik EB, Eren Y, Simavlı H, Uğurlu N, Çağıl N, et al., editors. Macula and retinal nerve fiber layer in migraine patients: analysis by spectral domain optic coherence tomography. Seminars in ophthalmology; 2015: Taylor & Francis.

72. Yurtoğulları Ş, Erbahçeci Timur İE, Eyidoğan D. Retinal Thickness Alterations in Patients with Migraine. Türk nöroloji dergisi. 2021;27(1):69-74.

73. Zengin MO, Elmas Z, Cinar E, Kucukerdonmez C. Choroidal thickness changes in patients with migraine. Acta Neurologica Belgica. 2015;115:33-7.
