# Supplementary material for: Using optical coherence tomography and optical coherence tomography angiography to delineate neurovascular homeostasis in migraine: a review
Source: Front Neurosci. 2024 Apr 15;18:1376282. doi: 10.3389/fnins.2024.1376282 (PMC11057254; doi:10.3389/fnins.2024.1376282)
Supplement: Supplementary file 2 [file Table_2.docx]

# Supplementary material 3

***Table 2.*** *Carroll diagram showing characteristic OCT/OCTA findings for each migraine subtype, compared to healthy controls.*

|  | **Episodic migraine** | **Chronic migraine** |
| --- | --- | --- |
| **Migraine without aura** | - Thinner RNFL, GCL and choroid (1-9) - More oxidative stress markers, less anti-oxidative molecular markers, especially in temporal quadrant (10) - Reduced retinal vessel and perfusion densities (11-14) - Larger FAZ (14) | - Thinner RNFL, choroid and GCL (3, 9, 15-17) - Thinner RNFL and GCL than episodic migraineurs (8, 9, 18-20) - Thicker choroid ictally (15) - Reduced macular retinal vessel and perfusion densities (11, 18) - Retinal artery diameters increased and choroidal thicknesses decreased ipsilaterally to headache (21) |
| **Migraine with aura** | - Even thinner RNFL and choroid than without aura (2-6, 8, 16, 22-24) - Reduced vessel and perfusion densities (11, 12, 25, 26), VD more so than without aura (27) - Larger FAZ (26, 27) - Thinner GCL (6) | - Thinner RNFL, choroid and GCL, more so than without aura (3, 8, 9, 15, 17, 19, 20, 28) - Larger FAZ (27) - Lower VD than without aura (27) - Thinner choroid, RNFL and GCL than without aura (16, 17) - Thicker choroid ictally (15) - Retinal artery diameters increased and choroidal thicknesses decreased ipsilaterally to headache (21) - Reduced retinal vessel and perfusion densities (11) - Reduced RNFL and GCL than episodic migraineurs (9) |

- 1. Acer S, Oğuzhanoğlu A, Çetin EN, Ongun N, Pekel G, Kaşıkçı A, et al. Ocular pulse amplitude and retina nerve fiber layer thickness in migraine patients without aura. BMC ophthalmology. 2016;16(1):1-8.
- 2. Demircan S, Ataş M, Arık Yüksel S, Ulusoy MD, Yuvacı İ, Arifoğlu HB, et al. The impact of migraine on posterior ocular structures. Journal of ophthalmology. 2015;2015.
- 3. Demirci S, Gunes A, Demirci S, Kutluhan S, Tok L, Tok O. The effect of cigarette smoking on retinal nerve fiber layer thickness in patients with migraine. Cutaneous and ocular toxicology. 2016;35(1):21-5.
- 4. Gipponi S, Scaroni N, Venturelli E, Forbice E, Rao R, Liberini P, et al. Reduction in retinal nerve fiber layer thickness in migraine patients. Neurological Sciences. 2013;34:841-5.
- 5. Gunes A, Demirci S, Tok L, Tok O, Demirci S, Kutluhan S. Is retinal nerve fiber layer thickness change related to headache lateralization in migraine? Korean Journal of Ophthalmology. 2016;30(2):134-9.
- 6. Kanar HS, Toz HT, Penbe A. Comparison of retinal nerve fiber layer, macular ganglion cell complex and choroidal thickness in patients with migraine with and without aura by using optical coherence tomography. Photodiagnosis and Photodynamic Therapy. 2021;34:102323.
- 7. Temel E, Aşikgarip N, Koçak Y, Şahin C, Özcan G, Kocamiş Ö, et al. Choroidal vascularity index and retinal nerve fiber layer reflectivity in newly diagnosed migraine patients. Photodiagnosis and Photodynamic Therapy. 2021;36:102531.
- 8. Yurtoğulları Ş, Erbahçeci Timur İE, Eyidoğan D. Retinal Thickness Alterations in Patients with Migraine. Türk nöroloji dergisi. 2021;27(1):69-74.
- 9. Reggio E, Chisari CG, Ferrigno G, Patti F, Donzuso G, Sciacca G, et al. Migraine causes retinal and choroidal structural changes: evaluation with ocular coherence tomography. Journal of neurology. 2017;264(3):494-502.
- 10. Bulboacă AE, Stănescu IC, Bolboacă SD, Bulboacă AC, Bodizs GI, Nicula CA. Retinal nerve fiber layer thickness and oxidative stress parameters in migraine patients without aura: a pilot study. Antioxidants. 2020;9(6):494.
- 11. He N, Shao H, He J, Zhang X, Ye D, Lv Z. Evaluation of retinal vessel and perfusion density in migraine patients by optical coherence tomography angiography. Photodiagnosis and Photodynamic Therapy. 2022;40:103060.
- 12. Ulusoy MO, Horasanlı B, Kal A. Retinal vascular density evaluation of migraine patients with and without aura and association with white matter hyperintensities. Acta Neurologica Belgica. 2019;119(3):411-7.
- 13. Güler Ö, Güler M, Tuğan Yıldız CB, Hakkoymaz H. Are Retinal and Peripapillary Blood Flows Affected during Migraine Attack? Neuroophthalmology. 2020;44(5):299-306.
- 14. Taşlı NG, Ersoy A. Altered Macular Vasculature in Migraine Patients without Aura: Is It Associated with Ocular Vasculature and White Matter Hyperintensities? Journal of Ophthalmology. 2020;2020.
- 15. Gunes A, Karadag AS, Yazgan S, Celik HU, Simsek A. Evaluation of retinal nerve fibre layer, ganglion cell layer and choroidal thickness with optical coherence tomography in migraine patients: a case‐control study. Clinical and Experimental Optometry. 2018;101(1):109-15.
- 16. Karaca EE, Koçer EB, Özdek Ş, Akçam HT, Ercan MB. Choroidal thickness measurements in migraine patients during attack-free period. Neurological Sciences. 2016;37(1):81-8.
- 17. Labib DM, Hegazy M, Esmat SM, Ali EAH, Talaat F. Retinal nerve fiber layer and ganglion cell layer changes using optical coherence tomography in patients with chronic migraine: a case-control study. The Egyptian Journal of Neurology, Psychiatry and Neurosurgery. 2020;56:1-6.
- 18. Oba T, Gulec ZEK, Cicek F, Uygunoglu U, Onder F. Retinal and peripapillary vascular density in episodic and chronic migraine cases without aura. Photodiagnosis and Photodynamic Therapy. 2023;44:103809.
- 19. Raga-Martínez I, Povedano-Montero FJ, Hernández-Gallego J, López-Muñoz F. Decrease Retinal Thickness in Patients with Chronic Migraine Evaluated by Optical Coherence Tomography. Diagnostics. 2022;13(1):5.
- 20. Sorkhabi R, Mostafaei S, Ahoor M, Talebi M. Evaluation of retinal nerve fiber layer thickness in migraine. Iranian journal of neurology. 2013;12(2):51.
- 21. Unlu M, Sevim DG, Gultekin M, Baydemir R, Karaca C, Oner A. Changes in retinal vessel diameters in migraine patients during attack-free period. International journal of ophthalmology. 2017;10(3):439.
- 22. Burgos-Blasco B, Ginés-Gallego C, Carrasco-López-Brea M, de Santos-Moreno MT, Santos-Bueso E. Retinal Nerve Fiber Layer Analysis in Children With Migraine With and Without Aura Using Optical Coherence Tomography: A Case–Control Study. Journal of Pediatric Ophthalmology and Strabismus. 2023;60(3):196-202.
- 23. Cankaya C, Tecellioglu M. Foveal thickness alterations in patients with migraine. Medical Archives. 2016;70(2):123.
- 24. El-Shazly AAE-F, Farweez YA, Hamdi MM, El-Sherbiny NE. Pattern Visual Evoked Potential, Pattern Electroretinogram, and Retinal Nerve Fiber Layer Thickness in Patients with Migraine during and after Aura. Current eye research. 2017;42(9):1327-32.
- 25. Kızıltunç PB, Atilla H. Vascular changes with optical coherence tomography angiography during aura of migraine: A case report. European Journal of Ophthalmology. 2020:1120672119899900.
- 26. Hamamci M, Songur MS, Bayhan SA, Bayhan HA. Is ocular vascularity affected in young migraine patients? A pilot study. Journal of Clinical Neuroscience. 2021;91:144-51.
- 27. Romozzi M, Cuffaro G, Rollo E, Mattei R, Marcelli S, Rizzo S, et al. Microvascular involvement in migraine: an optical coherence tomography angiography study. Journal of Neurology. 2023.
- 28. Colak HN, Kantarcı FA, Tatar MG, Eryilmaz M, Uslu H, Goker H, et al. Retinal nerve fiber layer, ganglion cell complex, and choroidal thicknesses in migraine. Arquivos brasileiros de oftalmologia. 2016;79:78-81.
