# Supplementary material for: Using optical coherence tomography and optical coherence tomography angiography to delineate neurovascular homeostasis in migraine: a review
Source: Front Neurosci. 2024 Apr 15;18:1376282. doi: 10.3389/fnins.2024.1376282 (PMC11057254; doi:10.3389/fnins.2024.1376282)

Supplementary material 2

Fig. 1. *Flowchart showing the number of studies per time course (episodic vs. chronic), presence or absence of aura, and study design (cross-sectional vs. longitudinal).*

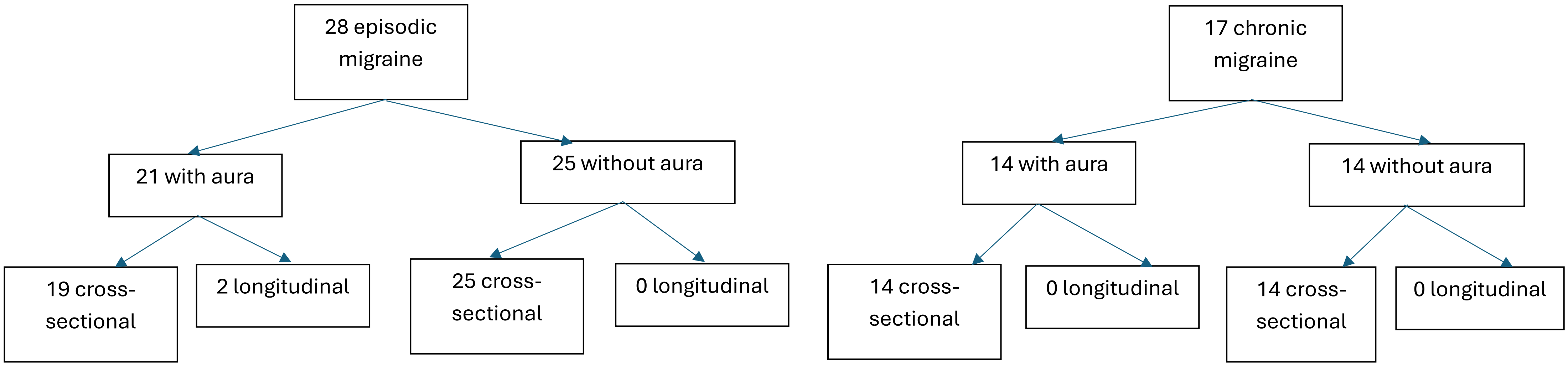

Supplement: Supplementary file 3 [file Image_1.pdf]
